# Oral Delivery of miR-320-3p with Lipidic Aminoglycoside Derivatives at Mid-Lactation Alters miR-320-3p Endogenous Levels in the Gut and Brain of Adult Rats According to Early or Regular Weaning

**DOI:** 10.3390/ijms24010191

**Published:** 2022-12-22

**Authors:** Gabriel Araujo Tavares, Amada Torres, Gwenola Le Drean, Maïwenn Queignec, Blandine Castellano, Laurent Tesson, Séverine Remy, Ignacio Anegon, Bruno Pitard, Bertrand Kaeffer

**Affiliations:** 1 Nantes Université, INRAE, UMR 1280, PhAN, F-44000 Nantes, France; 2 Laboratory of Neuroplasticity and Behavior, Graduate Program of Nutrition, Federal University of Pernambuco, Recife 56070-901, Brazil; 3 Platform Rat Transgenesis ImmunoPhenomic, INSERM UMR 1064-CRTI, SFR François Bonamy, CNRS UMS3556, F-44093 Nantes, France; 4 Nantes Université, Univ Angers, INSERM, CNRS, Immunology and New Concepts in Immunotherapy, INCIT UMR1302/EMR6001, F-44000 Nantes, France

**Keywords:** miRNA, neonatology, breast milk

## Abstract

To investigate if the artificial delivery of microRNAs naturally present in the breastmilk can impact the gut and brain of young rats according to weaning. Animals from a new transgenic rat line expressing the green-fluorescent protein in the endocrine lineage (cholecystokinin expressing cells) received a single oral bolus of miR-320-3p or miR-375-3p embedded in DiOleyl-Succinyl-Paromomycin (DOSP) on D-12. The pups were weaned early (D-15), or regularly (D-30). The expression of relevant miRNA, mRNAs, chromatin complexes, and duodenal cell density were assessed at 8 h post-inoculation and on D-45. The miR-320-3p/DOSP induced immediate effects on H3K4me3 chromatin complexes with *polr3d* promoter (*p* < 0.05). On regular weaning, on D-45, miR-320-3p and 375-3p were found to be downregulated in the stomach and upregulated in the hypothalamus (*p* < 0.001), whereas miR-320-3p was upregulated in the duodenum. After early weaning, miR-320-3p and miR-375-3p were downregulated in the stomach and the duodenum, but upregulated in the hypothalamus and the hippocampus. Combination of miR-320-3p/DOSP with early weaning enhanced miR-320-3p and chromogranin A expression in the duodenum. In the female brain stem, miR-320-3p, miR-504, and miR-16-5p levels were all upregulated. Investigating the oral miRNA-320-3p loads in the duodenal cell lineage paved the way for designing new therapeutics to avoid unexpected long-term impacts on the brain.

## 1. Introduction

In recent years considerable evidence has demonstrated that adult health status may be strongly influenced by experiences in early life modulated by epigenetic changes [1,2]. Inadequate, or an interruption of, lactation (early weaning) impairs an important nutritional and maternal contact, thereby promoting anxiety, depression, or stress in neonates, some with deleterious lifelong consequences. miR-504 and miR-16-5p play important roles in the relationship between early life stress and the modulation of the dopaminergic and serotonergic systems [3]. miR-504 directly targets the 3′-UTR of the dopamine D1 receptor gene (*drd1*) [4], whereas miR-16-5p is involved in the regulation of the serotonin transporter (*sert*) in the raphe of depressed rats [5]. Moreover, miR-132-3p has been described in neural cell epigenetics [6], where it couples circadian rhythms and the daily rhythms of neuron plasticity involved in cognition [7]. Although only a few changes in miRNA expression were reported after maternal separation, these studies support the hypothesis that early life stress induces susceptibility to later life stress at the epigenome level.

Moreover, the absorption of miRNAs in the rat stomach has been demonstrated to be regulated by systemic RNA interference–deficient transporter (*sidt1*) [8], indicating the possibility of the natural transit of miRNAs present in breastmilk from mother to offspring. Milk contains high levels of miRNAs that have been proposed to transfer between mother and child for immune regulation [9], thereby priming the immune system of the lactating infant (when the miRNAs are of plant origin) [10], and promoting transgenerational health [11] or the trans-species effect on adult consumers through dairy products [12,13]. Therefore, manipulating the physiopathology of rat pups with supplementation of extracellular miRNAs may integrate new knowledge for preventing the onset of chronic pathology at the earliest time possible. Although the potential of the oral delivery of extracellular miRNA has been revealed by several studies [14,15], its immediate and long-term effects on the molecular phenotype of a model organism and its consequences in the backdrop of early-life stress remain elusive.

Here, we focused on two microRNAs, miR-320-3p and miR-375-3p, common to rat and human breast milk. miR-320-3p is a non-canonical miRNA with a non-described 5p form in humans and rats [16], associated with breastmilk exosomes [17], and a highly conserved miRNA among mammals [18]. A *cis*-regulatory role is known for miR-320-3p, which participates in a negative feedback loop at the *polr3d* promoter, thereby inducing transcriptional gene silencing in Human Embryonic Kidney-293 cells [19]. In rat pups, we have reported an immediate effect of orally administered miR-320-3p on *hspb6* and *polr3d* mRNAs, and on *polr3d* promoter in chromatin complexes [20]. miR-320-3p is known for its bioactivity in various diseases such as type 2 diabetes, inflammatory bowel disease, and atherosclerosis [21,22,23,24]. miR-375-3p is one of the most abundant miRNAs in the gastrointestinal tract, impacting the homeostasis of the enteroendocrine lineage of mucosal cells [25], but without any reported activity in chromatin complexes. It has been associated with depression in children [26] and the differentiation of mouse neurites in the hippocampus [27]. In the bioprocessing of true miRNAs like miR-375-3p, both the 3p and 5p molecules are expressed allowing quantitative exploration by northern blot of their ratio in human cell lines [28]. This miRNA has been speculated to be involved in neuroprotective mechanisms in response to stress [29,30] and has further been shown to be associated with Alzheimer’s disease [31].

Therefore, in the present study, we aimed to investigate the effects of the artificial delivery of microRNAs naturally present in breastmilk on the gut and brain of young rats following early life stress. For their delivery, we used previously developed lipidic derivatives of natural aminoglycosides—allowed in food—that have been shown to efficiently deliver siRNA, DNA, mRNA, or miRNA to cells [20,32,33,34,35,36]. Lipidic aminoglycosides are used to embed RNA molecules diluted in a physiological buffer within minutes at room temperature, right before oral administration [20,35].

In this study, we demonstrate that force-feeding with miR-320-3p/DOSP at mid-lactation induced long-term effects on the gastrointestinal epithelium and brain of young rats, thereby deeply altering the regulation of endogenous miR-320-3p and miR-375-3p.

## 2. Results

### 2.1. In Silico Analysis of rno-miR-320-3p and rno-miR-375-3p Networks

The sequence coding for mature miR-320-3p was identical in rats, mice, and humans. Pairwise alignment revealed that miR-320-3p is antisense, encoded in the intergenic region of chromosome 8 at approximately 200 bp upstream of the Transcription Starting Site of RNA polymerase III subunit D (polr3d) in the rat (rn6), in chromosome 15 in the human (GRCh38.p13), and chromosome 14 in the mouse genomes [19]. We have found that the sequence coding for mature miR-375-3p was identical in rat, mouse, and human genomes. The mature full sequence of miR-375-3p was located on chromosome 9 for rats, chromosome 2 for humans, and chromosome 1 for mice. In the three genomes, it is located in non-coding regions.

Current miRNA target prediction algorithms regularly present different numbers of potential interactions. Therefore, we combined the results of three databases to obtain the most accurate list of target genes. For miR-320-3p and miR-375-3p, 111 and 24 transcripts distributed between 69 and 12 pathways, respectively, were identified (Supplementary Table S1). By the mining of miRWalk on 3′-UTR (with binding *p*-value set on 1.00), we identified 126 genes common between miR-320-3p and miR-375-3p, most of which corresponded to the KEGG pathway: rno01100_Metabolic_pathways. Furthermore, 308 and 112 genes were common between miR-320-3p and miR-132-3p, and miR-320-3p and miR-16-5p, respectively.

miRNA delivered to cells with appropriate carriers or expressed using suitable vectors often trigger both intended sequence-specific silencing effects and unintended sequence-non-specific immune responses [37]. Therefore, we selected the following inflammatory-related genes (GO:0006954_inflammatory_response and GO:0005125_cytokine_activity) to explore the inflammatory status of stomach samples: interleukin 1A (*IL-1A*), *IL-6*, interferon-gamma (*IFNγ*), signal transducer and activator of transcription 3 (*stat3*), *IL10*, tumor necrosis factor-alpha (*TNF-α*), *stat1*, *iNOS, ppar-γ* (peptide related to food consumption), *foxa1*, and *IL-1B*.

In brain samples, we explored the circadian locomotor output cycles kaput (*clock*) gene, which is common to miR-320-3p and miR-375-3p, along with brain and muscle ARNT-Like 1 (*bmal1*), Period1 (*per1*), and Period2 (*per2*). Interactions between miRNA and mRNA were built using miRWalk [38], and for the serotoninergic/dopaminergic profiles, we assessed *sert*, 5-hydroxytryptamine receptor 1B (*5ht1b*), 5-hydroxytryptamine receptor 2C (*5htr2c*), Dopamine receptor D1 (*drd1*), *drd2*, and Cholecystokinin (*cck*) genes. These genes are related to GO:0007420_brain_development, GO:0003676_nucleic_acid_binding, and GO:0006357_regulation_of_transcription_by_RNA_polymerase_II.

Furthermore, we assessed the markers of enteroendocrine lineage Paired box gene 4 *(pax4*), *pax6*, ghrelin (*ghrl*), Peptide YY (*pyy*), *chgA*, Gastric Inhibitory Polypeptide (*gip*), and *cck*.

**Figure 1:**
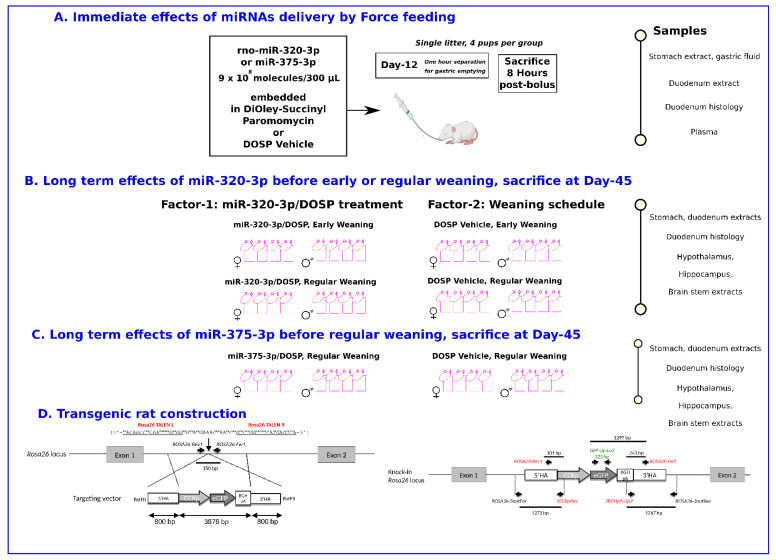
Study design. (**A**). Immediate effects of miRNA delivery. The rat pups at Day-12 (from one mother; *n* = 4 per group) were euthanized in the dark phase, 8 h after being fed with miR-320-3p/DOSP, miR-375-3p/DOSP, or the vehicle solution of DOSP. Samples were collected from the stomach and duodenum. (**B**). Long-term effects of miR-320-3p/DOSP given before early or regular weaning. Four litters were used, two for the early weaning schedule (at Day-15, miR-320-3p/DOSP or control), and two for the regular-weaning schedule (at Day-30, miR-320-3p/DOSP or control). Each litter has eight pups (four males, four females), treated with miR-320-3p/DOSP or the vehicle solution of DOSP at Day-12. All animals were euthanized at Day-45. The experiment allowed us to evaluate the miR-320/DOSP treatment (Factor-1), the weaning schedule (Factor-2), and the interaction between factors. Samples were collected from the stomach, duodenum, hypothalamus, hippocampus, and brain stem. (**C**). Long-term effects of miR-375-3p/DOSP given before regular weaning. Samples were collected from the stomach, duodenum, hypothalamus, hippocampus, and brain stem of eight rats from the same litter, treated with miR-375-3p/DOSP or the vehicle solution of DOSP at Day-12 and euthanized at Day-45. The second litter with eight rats receiving vehicle solution of DOSP and regular weaning was also done to check for differences with the first litter of rats treated with vehicle solution of DOSP at Day-12. In the gut, we have sampled the stomach as the inoculation site and the duodenum as relevant for our transgene expression. The rationale for choosing each brain area was for the hypothalamus as the main center of energy homeostasis, the hippocampus as involved in the memory of food reward and choice, and the brain stem as the outcome of the vague nerve linking the intestine to the brain. We have screened the effects according to gene sets related to the inflammatory and enteroendocrine status of the stomach or duodenum and on the serotoninergic/dopaminergic balance of brain areas. To illustrate the up- and downregulation of genes, our results were also presented as networks of miRNA and mRNA significantly deregulated in our dataset. Our transgenic GFP-CCK-p rat derived from the Sprague–Dawley strain allows us to follow an enteroendocrine cell lineage labeled with Green Fluorescent Protein in duodenum crypts. (**D**). Transgenic rat generation. Targeted integration of a CCK promoter-GFP cassette into the rat Rosa26 locus (**left panel**). Schematic representation of the rat Rosa26 locus. TALEN cleavage (vertical arrow) in the first intron, as well as the sequences recognized by both TALENs, the targeting vector with expression cassette (3878 bp), and the 5′ and 3′ homology arms (HA) (800 bp each), are indicated. The PCR primers flanking the cleavage sequence used to do the first genotyping are also indicated (**right panel**). Schematic representation of the CCK-GFP cassette integration. To verify the integrity of the CCK-GFP cassette, genomic DNAs were PCR amplified with the primers described and the PCR amplicons were analyzed for their size and Sanger sequences.

The experiments were organized to evaluate the immediate effects of force-feeding miRNAs/DOSP in the stomach and duodenum of breastfed pups on D-12 (Figure 1A), then the long-term effects of miR-320-3P/DOSP according to subsequent early or regular weaning (Figure 1B). In parallel, the long-term effects of miR-375-3p/DOSP treatment on rats submitted to regular weaning were obtained (Figure 1C). All experiments were realized on our indoors transgenic rats (Figure 1D).

### 2.2. Immediate Effects of Force-Feeding miRNAs/DOSP in the Stomach and Duodenum of Breastfed Pups on D-12 (Figure 1A)

The transgenic rat strain (Figure 1D) was checked for the correct expression of the transgene by PCR using tail biopsies to check for the homozygote status. Duodenal cross-section and immunostaining were realized on D-12 and D-45 rats to check for the expression of GFP-labeled duodenal cells according to the co-expression of *chgA*. The relative expression level of miR-375-3p and miR-320-3p (*p* = 0.056) increased in the stomach wall of rat pups fed with miR-320-3p/DOSP compared to that of rat pups fed with DOSP vehicle (Supplementary Figure S1A,B). Conversely, the *polr3d* tended to decrease, while *hspb6* showed a significant decrease in the miR-320-3p/DOSP group compared to that in the control group (Supplementary Figure S1C,D, *p* < 0.05). The treatment with miR-320-3p/DOSP induced a significant decrease in chromatin complexes harboring the H3K4me3 tag and the polr3d promoter in gastric cells (Supplementary Figure S1E, *p* < 0.05). No immediate effect of miR-375-3p/DOSP was observed on polr3d and hspb6 transcripts or chromatin complexes (Supplementary Figure S1C–E). The levels of D-glucose did not differ among the groups, as shown by the following data: 135 mg D-Glucose/dL ± 10.9 (mean ± standard deviation) in rats treated with miR-320-3p, 131.2 ± 15 in rats treated with miR-375, and 127.2 ± 6.5 in control rats. Furthermore, the levels of miR-320-3p or miR-375-3p in plasma showed no significant differences in the three groups, as shown by the following data: in the miR-320-3p/DOSP group: 22.79 ± 0.72 (Average Cq ± standard error) and 29.57 ± 0.65, respectively; in the miR-375-3p/DOSP group: 21.99 ± 0.89 and 27.78 ± 1.34, respectively; in the control: 21.83 ± 1.04 and 28.38 ± 0.84, respectively.

However, with miR-320-3P/DOSP, *chgA* was highly downregulated (*p* < 0.0001, Supplementary Figure S2A) and the ghrl transcripts were downregulated when comparing miR-320-3p/DOSP with miR-375-3p/DOSP treatment (Supplementary Figure S2B). We could not detect an immediate effect of miR-320-3p/DOSP or miR-375-3p/DOSP treatments in the stomach on *IL10*, *fox1*, *TNF-alpha*, and *stat1* (Supplementary Figure S2C–F).

In summary, these data on the immediate effect of rno-miR-320-3p/DOSP confirm that the miRNA molecules are bioactive both in the cytoplasm and in chromatin complexes.

### 2.3. Evaluation of the Long-Term Effects Induced by miR-320-3p/DOSP, with Subsequent Early or Regular Weaning (Figure 1B)

**Figure 2:**
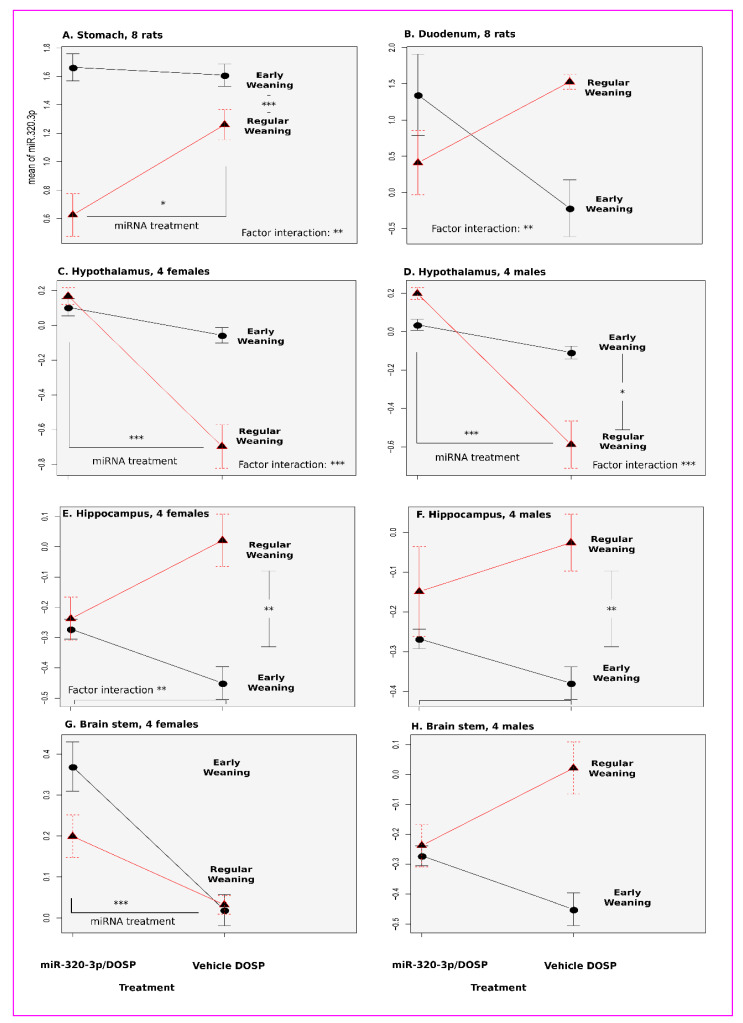
Long-term effects of miR-320-3p/DOSP treatment on miR-320-3p expression. The plot of means shows the expression of miR-320-3p in tissue extracts of the stomach (**A**), duodenum (**B**), hypothalamus (**C**,**D**), hippocampus (**E**,**F**), and brain stem (**G**,**H**). The miR-320-3p levels in brain stem extracts of female and male rats were strikingly different. A strong effect was found in the stomach but not in the duodenum. The light gray background indicates that rats were sacrificed in the dark phase. Note that the lines joining the points underline the direction of the variations, they are not curves of variations. * *p* < 0.05; ** *p* < 0.01; *** *p* < 0.001.

**Figure 3:**
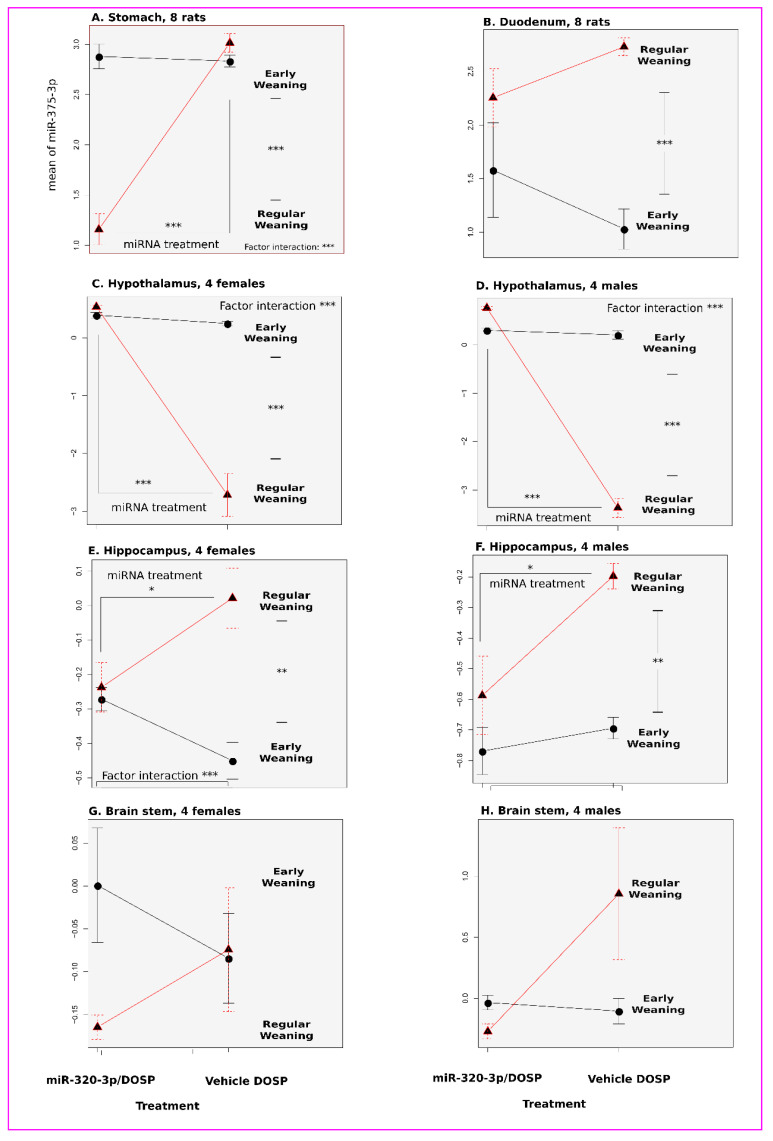
Long-term effects of miR-320-3p/DOSP treatment on miR-375-3p expression. The plot of means shows the expression of miR-375-3p in tissue extracts of the stomach (**A**), duodenum (**B**), hypothalamus (**C**,**D**), hippocampus (**E**,**F**), and brain stem (**G**,**H**). The miR-375-3p levels in brain stem extracts of female and male rats were strikingly different. A strong effect was found in the stomach but not in the duodenum. The light gray background indicates that rats were sacrificed in the dark phase. Note that the lines joining the points underline the direction of the variations, they are not curves of variations. * *p* < 0.05; ** *p* < 0.01; *** *p* < 0.001.

In the stomach, both factors (miR-320-3p/DOSP treatment and weaning schedule) were different for miR-320-3p (Figure 2A, *p* = 0.016, *p* = 0.0000009; respectively) as well as for miR-375-3p (Figure 3A, *p* = 0.00000001, *p* = 0.000000003; respectively). The interactions between factors for both miRNAs (weaning schedule and miR-320-3p treatment) were significant (*p* = 0.004 and *p* = 0.000000003, respectively).

**Figure 4:**
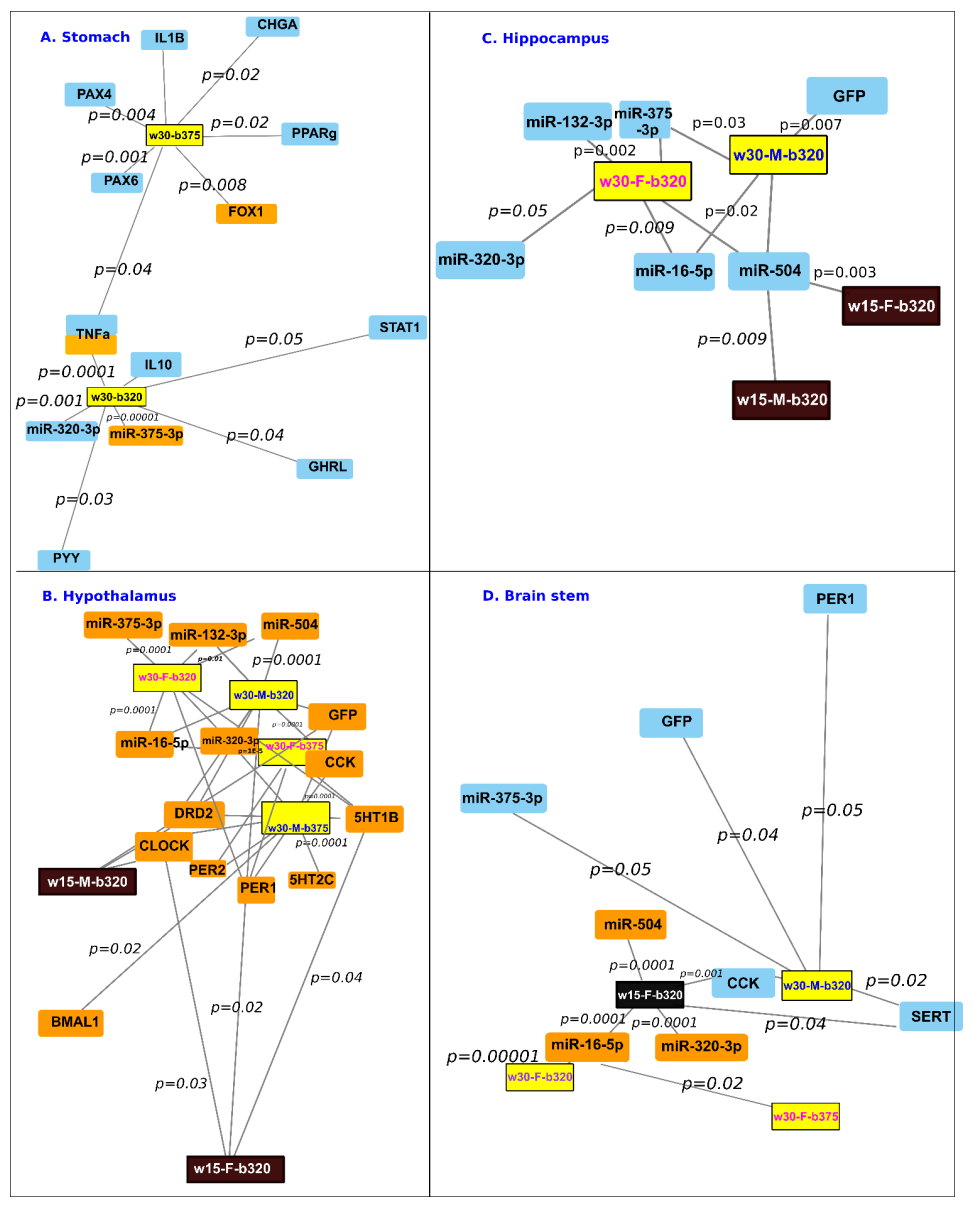
Network of genes significantly deregulated in the stomach wall (**A**), hypothalamus (**B**), hippocampus (**C**), and brain stem (**D**) for miR-320-3p/DOSP treatment according to early (w15) or regular (w30) weaning and of miR-375-3p/DOSP with regular weaning. Note the upregulation of miR-320-3p, miR-504, and miR-16-5p in the female brain stem. Edge length is inversely proportional to the *p* significant threshold. The *p* values are indicated in italic. Orange upregulated, blue downregulated.

The miR-320-3p and miR-375-3 relative levels of the early-weaning and control groups were not different (Figure 2A and Figure 3A), as well as for miR-16-5p (Mean Cq ± Standard Mean Error), of w15-b320 (19.83 ± 1.65) against w15-btem (20.16 ± 2.01). In contrast, miR-320-3p and miR-375-3p transcripts were, respectively, down- or upregulated after treatment with miR-320-3p/DOSP and regular weaning (Figure 4A).

We did not detect any difference between chromatin complexes harboring H3K4me3 and *polr3d* promoter in gastric cells after miR-320-3p/DOSP treatment and early or regular weaning (Supplementary Figure S1F,G). In addition, we did not find any difference for *sidt1* between early weaned rats treated with miR-320-3p/DOSP and corresponding controls (a trend of downregulation of *sidt1* was found in w15-b320 (32.55 ± 4.59) compared to that in w15-btem (26.19 ± 7.54). After miR-320-3p/DOSP with regular weaning, the downregulations of *grlh* (*p* = 0.04), *IL-10* (*p* = 0.00001), *foxa1* (*p* < 0.001), and *stat1* (*p* = 0.05) contrasted with the upregulation of TNF-α (*p* < 0.0001; Supplementary Figure S2B,F). The relative levels of *pyy* transcripts (*p* = 0.03) were downregulated.

Moreover, after early or regular weaning with or without treatments with miR-320-3p/DOSP or miR-375-3p/DOSP, we did not find any effect on *polr3d* and *hspb6* transcripts (Supplementary Figure S2G,H). Figure 4A displays a summary of all these data for the stomach extract.

In the duodenum (Figure 2B), both factors (miR-320-3p/DOSP treatment, weaning schedule) were not different for miR-320-3p. Interaction between factors was significant (Figure 2B, *p* = 0.003).

The levels of miR-375-3p (Figure 3B) and miR-375-5p were different only for the weaning schedule factor (*p* = 0.0002 and *p* = 0.0006, respectively). No interaction between factors for both miRNAs was found.

In the female hypothalamus, both factors (miR-320-3p/DOSP treatment and weaning schedule) were different for miR-320-3p (Figure 2C, *p* = 0.00002, *p* = 0.002; respectively) as well as for miR-375-3p (Figure 3C, *p* = 0.000001, *p* = 0.0000007; respectively). Interactions between factors for both miRNAs were significant (Figure 2C and Figure 3C, *p* = 0.0005 and *p* = 0.000003, respectively); likewise for miR-16-5p, miR-132-3p, and miR-504.

In the hypothalamus of early or regularly weaned females, the relative levels of all miRNAs are displayed Supplementary Figure S3A, as well as for other transcripts related to dopamine/serotonin (Supplementary Figure S5A), circadian clock (Supplementary Figure S6A) and cck, gfp (Supplementary Figure S7A). The 5ht1b (Supplementary Figure S5A), per1, and clock (Supplementary Figure S6A) transcripts are significatively upregulated.

In the male hypothalamus, both factors (miR-320-3p/DOSP treatment and weaning schedule) were different for miR-320-3p (Figure 2D, *p* = 0.00001, *p* = 0.03; respectively) as well as for miR-375-3p (Figure 3D, *p* = 0.01, *p* = 0.006; respectively). Interactions between factors for both miRNAs were significant (Figure 2D and Figure 3D, *p* = 0.0004 and *p* = 0.003, respectively); likewise for miR-16-5p, miR-132-3p, and miR-504.

In the hypothalamus of early or regularly weaned males, the relative levels of all miRNAs are displayed in Supplementary Figure S4A, as well as for other transcripts related to dopamine/serotonin (Supplementary Figure S5B), circadian clock (Supplementary Figure S6B) and *cck*, *gfp* (Supplementary Figure S7B). The early weaned males treated by miR-320-3p/DOSP displayed an upregulation of *drd2* (Supplementary Figure S5), *clock* (Supplementary Figure S6), and *gfp* (Supplementary Figure S7).

By contrast, the regularly weaned males treated by miR-320-3p/DOSP had an upregulation of *drd2* (Supplementary Figure S5), *per1* (Supplementary Figure S6), and *cck*, *gfp* (Supplementary Figure S7).

Figure 4B summarizes all significative upregulations for hypothalamus extracts according to sex, and early (w15) or regular weaning (w30). The relative levels of *clock* were upregulated in the hypothalamus of early weaned rats of both sexes. For early or regularly weaned males, the relative levels of *drd2* were upregulated.

In the female hippocampus, only the weaning schedule factor was different for miR-320-3p (Figure 2E, *p* = 0.002). Both miR-320-3P/DOSP treatment and weaning schedule factors were different for miR-375-3p (Figure 2E and Figure 3E, *p* = 0.01, *p* = 0.001; respectively). Interactions between factors for both miRNAs were significant (Figure 2E and Figure 3E, *p* = 0.005 and *p* = 0.002, respectively).

In the hippocampus of early or regularly weaned females, the relative levels of all miRNAs are displayed in Supplementary Figure S3B, as well as for other transcripts related to dopamine/serotonin (Supplementary Figure S5C), circadian clock (Supplementary Figure S6C), and *cck*, *gfp* (Supplementary Figure S7C). The females had a downregulation for miR-132-3p, miR-320-3p, and miR-375-3p (Supplementary Figure S3B).

In the male hippocampus, only the weaning schedule factor was different for miR-320-3p (Figure 2F, *p* = 0.006). Both miR-320-3P/DOSP treatment and weaning schedule factors were different for miR-375-3p (Figure 3F, *p* = 0.01, *p* = 0.001; respectively). No interactions were found between factors for both miRNAs.

Figure 4C summarizes all significative upregulations for hippocampus extracts according to sex and early (w15) or regular weaning (w30). Regularly weaned males had a downregulation of miR-375-3p, miR-16-5p, miR-504, and *gfp* transcripts. For early and regularly weaned rats of both sexes, the relative levels of miR-504 were downregulated (Figure 4C).

In the brain stem of female rats (Figure 2G and Figure 3G), the levels of miR-320-3p were different only for the miR-320-3p/DOSP treatment factor (Figure 2G, *p* = 0.0001). No difference was found with miR-375-3p (Figure 3G). No interactions were found between factors with the expression of miR-320-3p, miR-375-3p, and miR-16-5p, except with miR-504 and miR-132-3p (*p* = 0.001, *p* = 0.01, respectively).

In the brain stem of early weaned females, the relative levels of miR-320-3p, miR-16-5p, and miR-504 were upregulated (Supplementary Figure S3C), whereas the relative levels of *sert* and *cck* transcripts were downregulated (Supplementary Figures S6 and S7). A trend of upregulation was observed with miR-375-3p and miR-132-3p (Supplementary Figure S3C).

In the brain stem of regularly weaned females, the relative levels of miR-16-5p were upregulated (Supplementary Figure S3C).

In the male brain stem, no differences or interactions were found for miR-320-3p/DOSP treatment and weaning schedule on the levels of miR-320-3p and miR-375-3p (Figure 2H and Figure 3H).

The miR-320-3p level was upregulated, while the miR-132-3p transcript was downregulated significantly, as was the case for miR-16-5p expression (Supplementary Figure S4C).

*sert* and *cck* were downregulated in early weaned male rats (Figure 4D). The relative levels of *per1* (Supplementary Figure S6), and *cck*, *gfp* (Supplementary Figure S7) transcripts were significantly downregulated in male rats supplemented with miR-320-3p/DOSP and after regular weaning (*p* < 0.05). miR-504 was downregulated in all groups supplemented with miR-320-3p/DOSP, indicating a strong effect on this miRNA.

Figure 4D summarizes all significative up- or down-regulations for brain stem extracts according to sex and early (w15) or regular weaning (w30). The relative levels of *sert* and *cck* were downregulated in both early weaned male and female rats (Figure 4D).

Our transgenic rat expresses the *gfp* transcripts in all cells expressing CCK besides the enteroendocrine lineage of the duodenum. *gfp* was downregulated in young male rats after early or regular weaning treated with miR-320-3p/DOSP. A similar trend of *gfp* downregulation was observed in the hypothalamus of early weaned female rats treated with miR-320-3p. Moreover, the *gfp* level was altered in the hippocampus and brain stem of male rats supplemented with miR-320-3p/DOSP. No strong correlation between *cck* and *gfp* (coefficient = 0.537) was recorded.

In summary, long-term effects of oral exposure during lactation to the high concentration of miR-320-3p were found with unforeseen consequences on the transcript levels of endogenous miR-320-3p and miR-375-3p in the brain. The most striking result is about the upregulation of miR-320-3p in the duodenum, suggesting that the stem cell compartments along the gut were differently altered by the treatments with miR-320-3p/DOSP or with miR-375-3p/DOSP. Surprisingly, the early weaned male rats were more resilient to miRNA treatment, as their relative levels of miR-320-3p were already very high.

### 2.4. Effects of Early Weaning Comparatively to Regular Weaning (not Supplemented with miRNA)

**Figure 5:**
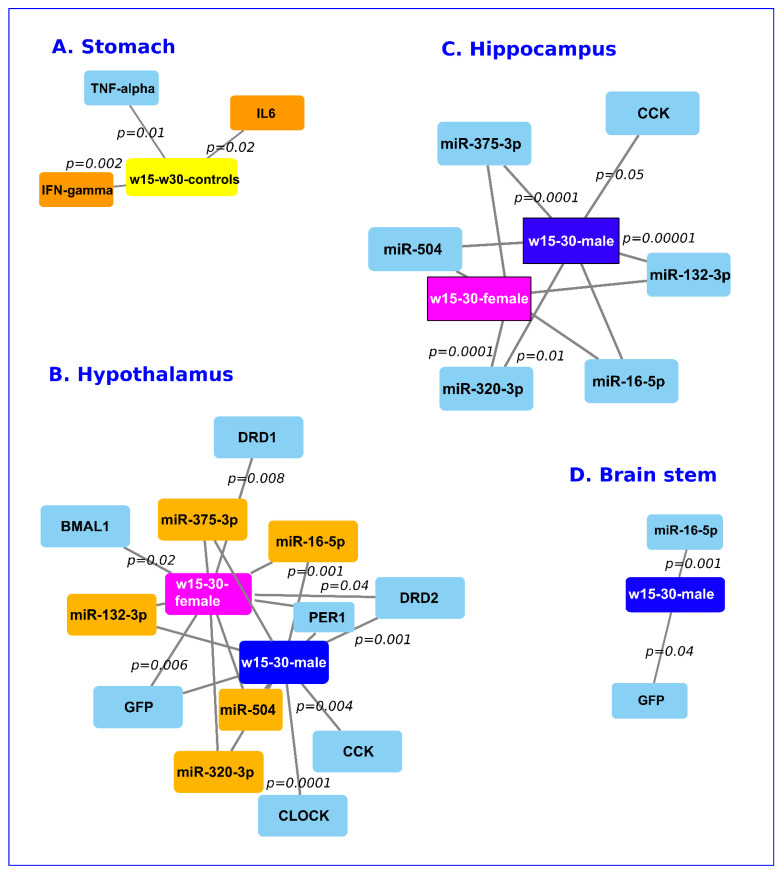
Network of genes significantly deregulated in the stomach wall (**A**), hypothalamus (**B**), hippocampus (**C**), and brain stem (**D**) of early and regularly weaned controls. Note on group nomenclature, for instance, “w15–w30” means a comparison between the early weaned controls with the regular weaning controls. No difference was found in the brain stem of female rats according to weaning schedule, and only miR-16-5p and gpf were downregulated in the brain stem of male rats. Edge length is inversely proportional to the *p* significant threshold. The *p* values are indicated in italic. Orange upregulated, blue downregulated.

In the stomach, the relative levels of chromatin complexes harboring H3K4me3 were slightly lower in the early weaned rats (Supplementary Figure S1F,G). Gastric endogenous miR-320-3p and miR-375-3p were not significantly different between early weaned and regularly weaned rats on D-45 (Figure 2A, Figure 3A and Figure 5A). Note that the miR-132-3p or miR-504 were not detected in the stomach samples. No difference between weaning times was observed for the expression of *polr3d* or *hspb6* mRNA (Supplementary Figure S2A,B). *TNF-α* was downregulated (Supplementary Figure S2E), and *IL-6* and *IFN-γ* were upregulated in early weaned controls compared to the regularly weaned controls (Figure 5A).

In the duodenum, endogenous miR-320-3p and miR-375-3p on D-45 were downregulated in early weaned rats (*p* < 0.01 and *p* < 0.05, respectively) compared to those in regularly weaned ones (Figure 2B and Figure 3B).

**Figure 6:**
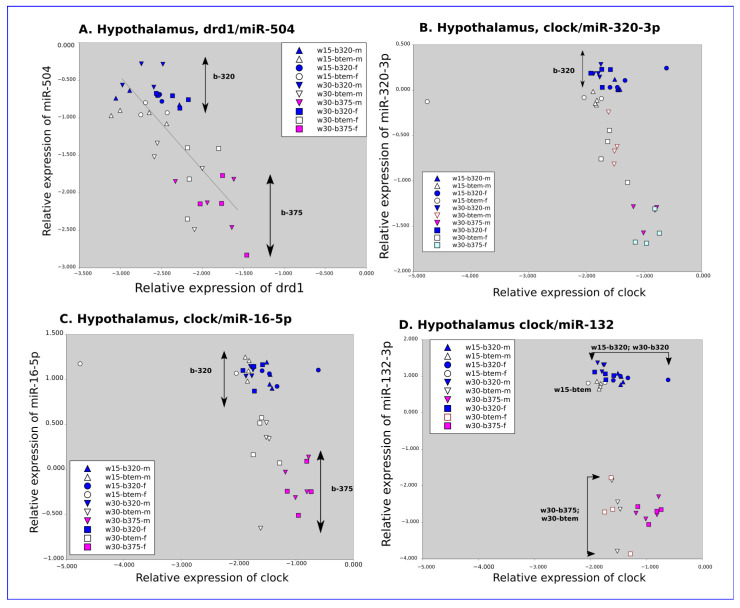
Long-term effects of miR-320-3p/DOSP according to early or regular weaning. Note the negative correlation (R = −0.75, linear regression in black) between miR-504 and DRD1 transcripts (**A**), and the upregulation of miR-320-3p (**B**), miR-16-5p (**C**) and 132-3p (**D**) for early weaned rats and regularly weaned rats treated by miR-320-3p/DOSP in hypothalamus cell extracts. indicates that rats were sacrificed in the dark phase.

In the hypothalamus of both female and male rats, all tested miRNAs (miR-320-3p, miR-375-3p, miR-16-5p, miR-132-3p, and miR-540) were upregulated (Figure 5B). However, we did not find any difference in the relative expression of *drd1* in the hypothalamus of male rats according to weaning time, even if an upregulation of miR-504 was recorded (Figure 5B, Supplementary Figure S4A). In summary, all tested miRNAs (miR-504, miR-16-5p, miR-132-3p, miR-320-3p, and miR-375-3p) were upregulated in the hypothalamus of early weaned rats on D-45. The *per1* transcripts of the circadian clock were downregulated in male and female rats (Figure 5B; Supplementary Figure S6A,B). However, *bmal1* was downregulated in females and *clock* was downregulated in males (Figure 5B; Supplementary Figure S6A). Additionally, *cck* and *gfp* were downregulated in the hypothalamus of early weaned rats. No strong correlation between *cck* and *gfp* (coefficient 0.537) was recorded (Supplementary Figure S7). A negative correlation (R = −0.75) between miR-504 and *drd1* transcripts has been found (Figure 6A). However, no correlation was obtained between miR-320-3p, miR-16-5p, and miR-132-3p for early weaned rats and regularly weaned rats treated by miR-320-3p/DOSP in hypothalamus cell extracts (Figure 6B–D). The levels of *clock* transcript were significantly different only in Figure 6B,C.

In the hippocampus, all tested miRNAs and *cck* were downregulated in both males and females (Figure 5C, Supplementary Figure S7). Only miR-16-5p and *gfp* were downregulated in the brain stem of male rats (Figure 5D).

### 2.5. Long-Term Effects Induced by miR-375-3p/DOSP, with Subsequent Regular Weaning (Figure 1C)

**Figure 7:**
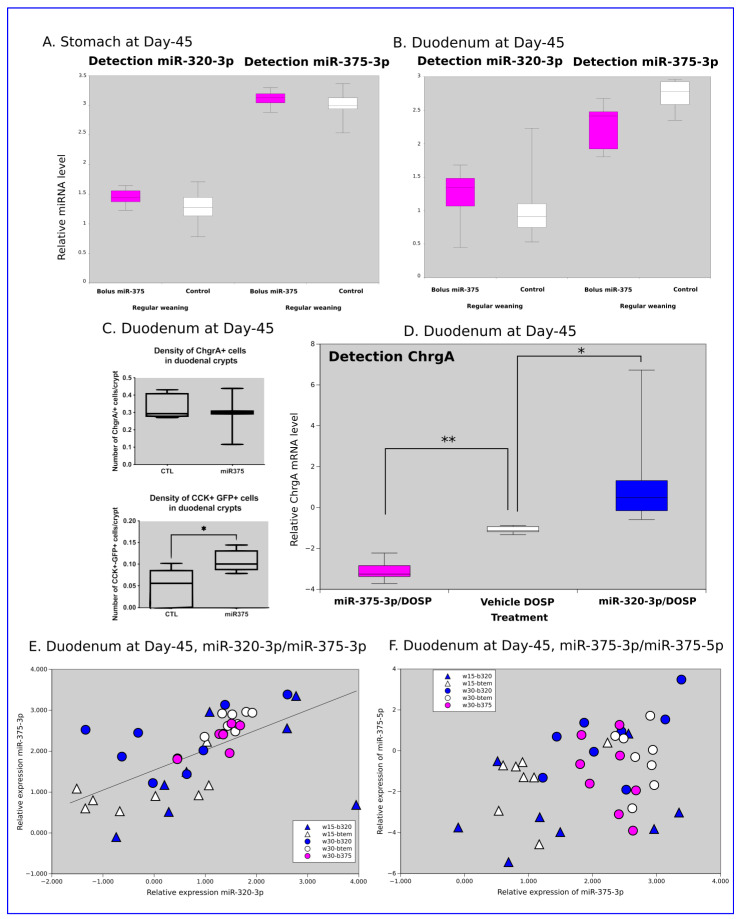
Long-term effects of miR-375-3p/DOSP followed by regular weaning on the density of CCK+GFP+ duodenal cells (**A**, *p* < 0.05), on the level of chromogranin A transcripts (**B**, *p* < 0.01). No difference was observed in miR-320-3p and miR-375-3p expressions in the stomach (**C**) and duodenum (**D**). Scatter plots between miR-320-3p/miR-375-3p and miR-375-3p/miR-375-5p are shown in (**E**) and (**F**), respectively. In (**E**), low correlation (R^2^ = 0.54, black line), and in (**F**), the miR-375-5p levels of w15-b320 were downregulated in comparison with w30-b320 (*p* = 0.02), and w30-btem (*p* = 0.04). The light gray background indicates that rats were sacrificed in the dark phase. Note: * *p* < 0.05; ** *p* < 0.01.

Neither miR-375-3p nor miR-320-3p showed changes in expression in rat pups force-fed with miR-375-3p/DOSP compared to those fed with DOSP vehicle in the stomach (Figure 7A) and duodenum (Figure 7B). However, the gastric relative levels of *chga*, *pparg*, *IL10*, *pax4*, *pax6*, and *TNG-alpha* were downregulated, and foxa was upregulated (Figure 4A).

In the duodenum, the miR-375-3p/DOSP treatment decreased both *chgA* (Figure 7C, *p* = 0.01) and *gip* (*p* = 0.001). A long-term effect of miR-375-3p/DOSP was noted on the density of GFP-CCK-p-labeled duodenal cells (Figure 7D, *p* < 0.05). We did not find a strong correlation between the relative levels of miR-320-3p and miR-375-3p (Figure 7E), nor between miR-375-3p and -5p (Figure 7F). However, miR-375-5p was downregulated in w15-b320 (compared to w30-b320 (*p* = 0.02), and w30-btem (*p* = 0.04, Figure 7F)).

In the hypothalamus, the levels of *5ht1b*, and *per1* and *per2* were altered in both males and females treated with miR-375-3p/DOSP (Figure 4B, Supplementary Figures S5 and S6). However, the males had an upregulation of bmal1, clock (Supplementary Figure S6), *drd2*, *5ht2c* (Supplementary Figure S5), and miR-320-3p (Supplementary Figure S4A). The females had an upregulation of miR-16-5p (Figure 4B, Supplementary Figure S3A). No differences were found with the hippocampus extracts (Figure 4C). Only miR-16-5p was upregulated in females fed with miR-375-3p/DOSP (Figure 4D).

## 3. Discussion

Oral supplementation by miRNA-320-3p or miR-375-3p during lactation has long-term miRNA-specific consequences on the endogenous levels of corresponding miRNAs with a strong tissue-dependent memory. Combining miR-320-3p/DOSP treatment (Factor-1) with the weaning schedule (Factor-2, early or regular weaning) has shown a strong interaction between factors in all tissue extracts except the brain stem. Combining miR-320-3p/DOSP with early weaning enhanced miR-320-3p and *chgA* expression in the duodenum. In the hippocampus, the miR-504 was downregulated in both sexes, but in the brain stem, it was upregulated only in females, along with miR-320-3p and miR-16-5p levels. In the hypothalamus, *clock* was upregulated in both sexes (Table 1).

The miRNA/DOSP complexes are delivered in the stomach, but according to the described kinetics [20], they can also be delivered in proximal sites of the small intestine. Our transgenic rat model allowed us to explore the influence of miRNA supplementation at a distance from the inoculation site on the neuroendocrine cell lineage of the duodenum. Here we used miR-320-3p with cytoplasmic and nuclear sites of bioactivity, in parallel with miR-375-3p with bioactivity limited to the cytoplasm. The administration of DOSP loaded with a specific miRNA can be considered neutral for any physiological effects triggered by the miRNA. Even though paromomycin, the polar headgroup of DOSP, potentially targets the mammalian ribosome machinery [39], our current vector is bypassing the physiological *sidt1*-adsorption of miRNA in the stomach [8]. Likewise, we did not detect the loading of miR-320-3p or miR-375-3p in gastric extracellular vesicles. The sequences of these miRNAs have no sorting sequences in exosomes [40], which favors the absence of the re-exporting of these miRNAs after their cytoplasmic delivery either toward the gastric lumen or into the blood. No immediate effect of miR-320-3p/DOSP is described in the plexus choroid or cortex [20]. Surprisingly, both miR-320-3P/DOSP and miR-375-3p treatments had an impact on the endogenous levels of these miRNAs in the brain. Our data indicate a very high variability of miR-320-3p detection after 8 h in the plasma of miR-320-3p/DOSP or miR-375-3p/DOSP groups compared to controls. It seems highly likely that DOSP complexes were able to get through the digestive epithelium into the plasma, then reach the brain-blood barriers. The DOSP delivers the RNA cargo in the cytoplasm of any cell, as demonstrated in vitro and on gastric glands maintained in culture [20]. The use of a transgenic rat is crucial in that respect, as green fluorescence-labeled epithelial cells of the enteroendocrine lineage are detected in the mature and proliferative compartment of the villus/crypt structure. We cannot label our DOSP vector for fluorescent tracking in blood and lymph, but DOSP could be tailored for targeting specific gut cell lineages and its putative interaction with the ribosome machinery. Such a vector would help resolve the paradox of a gut delivery with consequences in the brain area on the levels of endogenous miR-320-3p or miR-375-3p. The delivery of miRNAs with lipidic aminoglycosides can be added to the nanoparticles industrially available [41]; their main advantage over exosomes is that the loading is easy and reliable [36,42].

miR-320-3p is present in breast milk exosomes, but there is no report on any effect of this miRNA on the homeostasis of pups (being loaded in exosomes or lipoprotein complexes as reviewed by Groot and Lee (2020) [43]). Our data strongly support using these breastmilk miRNAs as supplements in lactating rat pups, even with discordant results on the stomach (non-significant effects for miR-320-3p or miR-375-3p in Figure 2A), and on the duodenum (significant effects for miR-320-3p in Figure 2B). The endogenous miR-320-3p in the duodenum and miR-320-3p and miR-375-3p in all brain compartments tested were altered in control rats subjected to early weaning (Figure 2, Figure 3, Figure 4 and Figure 5). However, our data revealed a downregulation of both miR-375-3p and miR-320-3p in the hippocampus of early weaned young rats in contradiction with the increased expression of miR-375-3p in the hippocampus of stressed mice [29].

Early postnatal life is a critical period where stressful experiences may potentially lead to long-term programming. The application of preventive and therapeutic approaches during early-life-sensitive periods is likely promising. For example, if the epigenetic patterns disrupted by exposure to stress can be modified through specific epigenome-targeted therapeutic interventions, it would be possible to correct the impaired gene expression patterns to prevent stress-induced chronic pathologies and improve human health and longevity. Tavares et al. (2020) inferred that miR-16-5p is an excellent candidate for moderating changes in *sert*, *5ht1a*, and *5ht2a* due to early life stress [3]. Table 1 shows that miR-16-5p and *sert* or *cck* are inversely regulated in the female brain stem. Thus, our results are in favor of long-lasting alterations of miR-504 and miR-16-5p in the brain stem depending on the sex. Sex plays a critical role in the brain according to previous works [3], which is consistent with those of McKibben et al. (2021) [44], who have found that in the hypothalamus, miR-132-3p, and miR-504 are responsive to early life stress, with males expressing greater changes following postnatal stress.

The levels of *drd1* were downregulated in the hypothalamus of early weaned females with a higher expression of miR-504, according to Huang and Li, 2009 [4]. We establish here a relationship between neonatal stress and the modulation of the serotonergic and dopaminergic systems, through post-transcriptional regulation by miRNAs, as a possible pathophysiological mechanism behind disorders induced by early weaning. Interestingly, miR-504 targets both *drd1* and *drd2*, with their expression being altered by early life stress [45]. Additionally, drd1 has also been identified as a target for miR-16 [46]. The early weaned rats were more resilient to miR-320-3p/DOSP treatment on the expression of endogenous miR-320-3p and miR-375-3p. The innate immunity of early weaned rats at the stomach level is also deeply altered, partly linked to the alteration in gastrointestinal permeability [47]. However, our treatment with miR-320-3p/DOSP did not induce significant evolution of the cytokines related to immunity. Immune dysregulation is a key pathway linking childhood adversity to elevated morbidity and mortality rates from several chronic diseases later in life. However, the double stress of miR-320-3p/DOSP and early weaning did not alter miR-375-3p and *sidt1* transcripts. The modification of *clock* transcripts in the hypothalamus or the liver of young Wistar rats on D-35 shows an increased level in the nocturnal situation [38]. Our data on transgenic Sprague–Dawley rats have been obtained with euthanasia done in the nocturnal phase (Figure 1A–C). However, despite in silico prediction, the miR-320-3p, -16-5p, and -132-3p did not correlate with *clock* transcripts (Figure 6B–D). On the contrary, in early weaned rats force-fed with miR-320-3p, *clock* and *per1* levels were high in the hypothalamus of males and females, respectively (Figure 4B, Table 1). Therefore, further experiments are needed to explore whether early weaning stress with or without force-feeding miR-320-3p alters the circadian clock machinery. Moreover, in Figure 4B, with early weaned rats force-fed with miR-320-3p, clock levels were high in the hypothalamus of males, and period1 in females (Table 1). Future work in the developmental biology domain of the circadian clock could open efficient therapeutic avenues.

As shown in Figure 4 and Figure 5, the networks of genes significantly deregulated in the stomach or brain compartments for early weaned rats are very narrow compared to the networks obtained after regular weaning. In the young rats raised with regular weaning, the supplementation with miR-320-3p/DOSP revealed a higher impact on targeted miRNAs than in early weaned rats. The miR-320-3p/DOSP supplementation also upregulated the endogenous miR-375-3p. In contrast, the effect of miR-375-3p/DOSP supplementation is weaker than that of miR-320-3p according to the limited subset of transcripts under the regulation of this miRNA. Furthermore, our results demonstrate an increased density of enteroendocrine GFP-labeled cells, suggesting that the concentration of miR-375-3p was high enough to be delivered in duodenal proliferative or stem cells with late consequences on the kinetics of the duodenum. No strong correlation between *cck* and *gfp* (coefficient = 0.537) was recorded, suggesting that even if the promoter was driven by the same transcriptional machinery as *cck*, the transgene was independently regulated from the *cck* endogenous gene promoter (Supplementary Figure S5). We confirm with a strain of Sprague–Dawley rat, the data of immediate effects obtained on the Wistar strain [20], using the same Day-12 for administration, corresponding to neuronal diversification [48]. Likewise, the *chgA* and *gip* transcripts were downregulated with supplementation of miR-375-3p/DOSP and upregulated with supplementation of miR-320-3p/DOSP, compared to supplementation of control bolus. The endogenous miR-320-3p on D-45 was upregulated in the duodenum (Figure 7D), indicating that the miR-320-3p/DOSP treatment can restore this miRNA level according to its relative expression level after regular weaning. These findings could assist in developing new therapeutic strategies to prevent early life stress by targeting duodenal enteroendocrine cells. However, further studies are required to explore the direct effects of miRNA supplementation on the stem cells of duodenal epithelia.

Before weaning, a single bolus of miRNAs induced a long-term effect on the expression of several miRNAs and mRNA in young rats, depending on the miRNA given by force feeding. In the stomach, the endogenous miR-320-3p and miR-375-3p (Figure 2A) were significantly lower in rat pups treated with miR-320-3p/DOSP compared to those treated with control bolus or miR-375-3p/DOSP. In all brain compartments tested, endogenous mir-320-3p and 375-3p were significantly upregulated in both male and female rat pups in the miR-320-3p/DOSP group compared to those in the control and miR-375-3p/DOSP groups (Figure 4 and Figure 5). The data revealed that force-feeding rat pups with miR-375-3p, targeting fewer genes than miR-320-3p, consequently delivered miR-375-3p without any effect on the endogenous level of miR-320-3p. This data favors a non-described hierarchical molecular link between endogenous miR-320-3p and miR-375-3p. Force-feeding rat pups with miR-320-3p/DOSP revealed that, as predicted with our in silico data showing a wider target range for this miRNA, the rat pups with regular weaning have deregulation in the stomach as well as in brain compartments, impacting endogenous miR-375-3p. In addition, miR-375-5p was expressed in duodenum extracts without any difference between early weaned rats treated with miR-320-3p/DOSP and controls. The miR-375-3p and 5p are both expressed in several rat tissue [49], but to our knowledge of the 5p form, without any description of their physiological effect. miR-375-3p is well-known for targeting the proliferation/differentiation in the intestine; however, its nuclear activity and interaction with miR-320-3p have not been explored. In this study, assessing both miRNAs together paved the possibility of comparing miRNAs with cytoplasmic and nuclear targets (miR-320-3p), and with cytoplasmic targets (miR-375-3p). Future research on a specific epithelial cell lineage is needed to explore the dynamic of the ratio between miR-375, 3p, and 5p molecules in single-cell. Young et al., 2022 have shown that stoichiometry exists between miR-140-5p and 140-3p with a physiological effect for cartilage biosynthesis [50]. Future work is needed in breastmilk supplementation by considering the risk of displacing the ratio between 5p and 3p for canonical miRNA like miR-375. To our knowledge, the incidence of a lower amount of miR-26a in mouse breastmilk has been reported with physiological consequences in the adipocyte compartment [51]. Yarani et al. (2022) have explored using meta-analysis the role of miRNAs in ulcerative colitis and Crohn’s Disease, underlining that the miR-375-3p is downregulated during the development of these inflammatory diseases [52]. Oral supplementation with miR-375-3p could open new therapeutic avenues for curing these diseases.

In therapeutics applications, miR-320-3p is currently being explored for its bioactivity in various diseases from type 2 diabetes to atherosclerosis [22,23,24]. The in vivo delivery of miR-320-3p is targeting binding sites located both on the *polr3d* promoter and on *polr3d* 3′-UTR. Polr3d is the subunit-17 of polymerase-III involved in tumorigenesis. RNA polymerase III is considered to be linked to aging and longevity through TORC and insulin genes, as well as through genes related to telomerase activity [53,54]. However, we have shown only an immediate effect on the chromatin complexes related to H3K4me3, as well as an absence of a long-term effect on *polr3d* mRNA. miR-320-3p has been studied for post-transcriptional gene silencing in the cytoplasm of rat endothelial and cardiac cell cultures derived from diabetes situations, on several genes, among which is the heat shock protein family B (small) member 6 [55]. The *hspb6* (also *hsp20*) gene is highly expressed in several organs including the stomach [56]. Our data confirmed the immediate effects on *polr3d* and *hspb6* genes [20]; however, additional work is needed to explore the putative long-term effects on the polr3d complex, which includes 17 subunits, as well as any effect on telomerase activity.

One limitation of our work is that the transcriptomic data need confirmation at the protein level for a full appreciation of the impact on the adaptive immunity of the host. A second limitation is a need for a behavioral study to explore the long-term consequences of preventive administration of miR-320-3p on the health of rats. Finally, our vector allows for the administration of a cocktail of several RNA molecules, which constitutes a promising field of future exploration in immunotherapy.

In summary, supplementations with miR-320-3p/DOSP or miR-375-3p/DOSP were more potent in the young rats raised with regular weaning. However, the early weaned male rats were more resilient to miRNA treatment as their relative levels of miR-320-3p were already very high. The miR-504 was unchanged in the hypothalamus, downregulated in the hippocampus, but upregulated along with miR-320-3p and miR-16-5p in females treated with miR-320-3p/DOSP.

In conclusion, supplementation of lactating rat pups with extracellular miR-320-3p given before early weaning stress alters the expression of miR-320-3p in the duodenum, miR-375-3p in the brain stem of females, and *clock* transcripts in the hypothalamus (Table 1), thereby highlighting the need for behavioral studies. We have described a new relationship between two unrelated miRNAs, miR-320-3p and miR-375-3p, underlining a hierarchy between miRNA networks. The exploration of the therapeutic potential of miRNAs needs an integrative physiology approach, with a highly specific site of delivery, like duodenal enteroendocrine cell lineage, and one that revolves around the competing endogenous RNA hypothesis [57]. This approach would gain momentum by implementing results in an international database, thereby reducing the gap between in silico prediction and biological observations. The development of a new milk formulation intended to manipulate the epigenetics of babies [58] will benefit from such preclinical models.

## 4. Materials and Methods

### 4.1. Animals

Nine litters of our transgenic rat strain corresponding to a total of 76 rat pups were obtained from UMR-1280 husbandry. Litters were housed in allocated rooms with a 12 h/12 h light/dark photoperiod. The lights were switched off at 7:00 a.m. and switched on at 7:00 p.m. After birth, the rat pups were separated from their mothers either on D-15 (early weaning) or D-30 (regular weaning) and were fed soup made from standard chow. Our experimental protocol was approved by the “Comité d’éthique pour l’expérimentation animale, Pays de la Loire, France” (Approval number #APAFIS-21917). Studies on rats were performed according to the rules of the Nantes animal experimental unit [in compliance with the European Communities Directive of 2010/63/UE, 22 September 2010].

### 4.2. miRNAs

Two miRNAs present in breast milk were used for oral supplementation, i.e., miR-320-3p (MIMAT0000903 AAAAGCUGGGUUGAGAGGGCGA) already described by us and others as having epigenetic activity [19,20], and miR-375-3p MIMAT0005307 UUUGUUCGUUCGGCUCGCGUGA, known to target proliferative cells in the gut and related to vitamin-E metabolism in humans [59]. rno-miR-320-3p or rno-miR-375-3p were procured from Eurofinns, Germany, and they were verified upon acquisition (by reverse transcribing cDNA with TaqMan miRNA kit and q-PCR detection with corresponding TaqMan probes, Supplementary Table S2).

### 4.3. miR-320-3p and miR-375-3p Target Enrichment Analysis

TargetScan 7.2 (date: March 2018) (http://www.targetscan.org; accessed on 1 March 2018), miRWalk v6.0, (update: 20 January 2021 www.umm.uni-heidelberg.de/apps/zmf/mirwalk; accessed on 20 January 2021), and miRDB v6.0 (update: June 2019, http://mirdb.org/; accessed on 1 June 2019) were used to identify potential target genes regulated by miR-320-3p or miR-375-3p by exploring the miRNA binding sites within the complete sequence of the rat genome (including 5′-UTR, 3′-UTR, and coding sequences), and then combining this information for a comparative analysis of the predicted binding sites. The three databases were jointly mined, and overlaps of the results were generated to obtain the list of the transcripts most potentially regulated.

### 4.4. miR-320-3p and miR-375-3p Pathway Enrichment Analysis

To identify pathways in the list of the potentially regulated mRNAs, we used Panther v16.0 (released 1 December 2020, http://www.pantherdb.org/). To ensure the validity of our findings, we only considered the three pathways most relevant to both miRNAs.

### 4.5. Ribonucleic Acid Vector

Di-oleyl-succinyl-paromomycin (DOSP) vector was used for in vivo short-term transfection of miRNA [20], kindly provided by In-Cell-Art (Nantes, France). The vector is non-cytotoxic [20] and is allowed in food practice. Before use, the quality of the vector was assessed by the size distribution of DOSP nanoparticles with a peak at 200 nm on a Gold-q-Nano (Izon, Lyon, France).

### 4.6. Study Design

A scheme of our study design is shown in Figure 1A–C. The first litter (from one mother) with 12 rat pups was used to assess the immediate effect of miRNAs. The pups were divided into three groups (*n* = 4 per group) by randomly assigning them to one of the following treatments: miR-320-3p/DOSP, miR-375-3p/DOSP, and control (vehicle solution of DOSP), and identified by using an indelible marker on head and legs (Figure 1A). After separation from their mother, the pups were maintained at a warm temperature (37 °C) in a parallel transparent box next to their mother box for 1 h for gastric emptying. On D-12 at light off, the pups in the treatment groups were force-fed either miR-320-3p or miR-375-3p embedded in DOSP, referred to later as miR-320-3p/DOSP and miR-375-3p/DOSP groups, respectively. The pups in the control group were force-fed with the vehicle solution of DOSP. For oral inoculation, all solutions of deep-frozen miRNAs and DOSP incubated at 4 °C were warmed to room temperature, and the mixtures of miRNAs and DOSP were extemporaneously prepared. Force-feeding was gently performed at the beginning of the dark phase by trained experimenters, allowing the delivery of 9 × 10^8^ miRNA molecules in the stomach [20]. To assess the immediate effect of the miRNAs, the pups (from one mother; *n* = 4 per group) were euthanized in the dark phase, 8 h after being fed the supplements.

The long-term effects of miR-320-3p were assessed on eight rats from the same litter (four males, four females), treated with miR-320-3p/DOSP or vehicle solution of DOSP at Day-12, then submitted to early weaning schedule or regular weaning (Figure 1B). All animals were euthanized at Day-45 [60]. The experiment allowed us to evaluate, the miR-320/DOSP treatment (Factor-1), the weaning schedule (Factor-2), and interactions.

In parallel, the long-term effects of miR-375-3p were assessed on eight rats from the same litter (four males, four females), treated with miR-375-3p/DOSP at Day-12, submitted to regular weaning or vehicle solution of DOSP at Day-12 and euthanized at Day-45 (Figure 1C). The second litter with eight rats (four males, four females) receiving vehicle solution of DOSP and regular weaning was also done to check for differences with the first litter of rats treated with vehicle solution of DOSP at Day-12. The pups in these treatment groups and their respective controls were euthanized on D-45 after force-feeding.

Two additional litters were weaned at D-15 with pellets instead of soup, as usually done with the Wistar strain [61]. However, the Sprague–Dawley pups were unable to adapt to dry pellet, and the results obtained with them were not included in our analyses. We strongly recommend early weaning of Sprague–Dawley pups with soup made from standard chow.

### 4.7. Development of Transgenic GFP-CCK-p Rat

As shown in Figure 1D, Sprague–Dawley transgenic rats expressing enhanced GFP (eGFP) under the control of the CCK promotor were generated using the transcription activator-like effector nuclease (TALEN) methodology, and those with knock-in events in the *Rosa26* locus [62] were selected. This transgenic rat line was used to investigate CCK-eGFP^+^ cells in duodenal crypts and villi [63]. In addition, the eGFP transcripts were identified in rat neuronal cells expressing CCK [64]. qPCR was used for genotyping, and the qPCR primers are shown in Supplementary Table S2.

### 4.8. Gastric Fluids and Tissue Samples

Gastric fluids were collected to evaluate a putative re-export of loaded miRNA through extracellular vesicles, 8 h after administration. Briefly, at euthanization, the stomach contents were collected and stored in liquid nitrogen. Before analysis, the samples were thawed, and extracellular vesicles were recovered by elution through a qEV column (Izon) and processed for size distribution analysis with a qNano system (Izon). The immediate effect of the miRNA/DOSP bolus was also evaluated by measuring the D-glucose levels in the blood, and the contents of miR-320-3p and miR-375-3p in the exosome fractions of gastric fluids and rat pup plasma. For immediate (8 h) and long-term (D-45) studies, the stomach and the duodenum were rinsed with calcium- and magnesium-ion-free phosphate-buffered saline (PBS). Pieces of the lower part of the stomach (fundus) and duodenum were immersed in liquid nitrogen. In addition, pieces of the duodenum were fixed in 4% paraformaldehyde prepared in 0.1 M PBS for 24 h and then embedded in paraffin for histology. In addition to the stomach and duodenum, the brain compartments of the rat pups euthanized on D-45 were harvested and immersed directly in liquid nitrogen.

### 4.9. Analysis of miRNA and mRNA by q-PCR

For gene expression analysis, all organs were homogenized in QIAzol buffer (Qiagen, Germany) using Precellys tissue homogenizer (Bertin Technologies, Montigny-le-Bretonneux, France). Total RNA was extracted with QIAzol (Qiagen), and cDNA was obtained using a TaqMan miRNA kit (Thermo Fisher, Saint-Herblain, France). Real-time quantitative PCR was performed using a Bio-Rad CFX connect real-time system (Hercules, CA, USA) in a final volume of 10 µL.

miRNA expression was evaluated in accordance with the protocol proposed by Beuzelin et al., 2019 [20], using three miRNAs as reference genes (rno-miR-146b-5p, let-7d-5p, and let-7g-5p). Likewise, mRNA expression was evaluated using three reference genes (rno-β-2-microglobulin, β-actin, and Usb [20]. We have taken into account criticisms of normalization against reference genes [65] by checking the stability of our reference genes. All primers are listed in Supplementary Table S2. The primers used were either TaqMan primers or self-designed primers for use with SYBR Green.

### 4.10. Chromatin Immunoprecipitation

Methylation of Histone 3 at lysine residue 4 is generally associated with the transcriptional activation of nearby genes; however, little is known about the nuclear delivery of the non-complexed miRNA [66,67]. Here, we performed chromatin immunoprecipitation using Pierce Chromatin Prep Module (Thermo Fisher Scientific #26158) in accordance with the procedure described in [20]. Briefly, small tissue aliquots were crosslinked using 1% formaldehyde. Chromatin was fragmented using ChIP-grade micrococcus nuclease from the kit (0.25 μL/sample), and immunoprecipitation was performed by overnight incubation with 1 μg anti-trimethyl-Histone-3-Lys-4 antibody (Thermo Fisher Scientific; catalog# PA5-17420) at 4 °C.

We collected immune complexes by incubating with agarose A/G beads for 2 h at 4 °C; beads were rinsed twice with PBS and pelleted at 94× *g* for 1 min. Immune complexes were eluted using 100 μL elution buffer. After brief vortexing, preparations were incubated at room temperature for 15 min. Thereafter, beads were spun down at 94× *g* for 1 min, and the supernatant (eluate) was transferred to a fresh tube. The elution step was repeated. Both eluates were combined.

Then, 5 M NaCl and proteinase K were added to the eluate and the mixture was incubated for 1.5 h at 65 °C to reverse the crosslinking. Subsequently, nucleic acids were recovered by Qiagen miRNA-Easy kit, and ChIPped chromatin was analyzed using quantitative PCR (performed using iQ SYBR Green Supermix (Bio-Rad) on an iCycler iQ system (Bio-Rad), with promoter-specific primers) (Supplementary Table S2).

### 4.11. Density of Duodenal Green Fluorescence-Labeled CCK-p Enteroendocrine Cells

Following rehydration, 4 µm thick sections were stained with rabbit polyclonal chromogranin A (chgA; a marker of total enteroendocrine cells) antibody (diluted 1/500; 20085; Immunostar, Hudson, WI, USA). After incubation with a secondary biotinylated goat anti-rabbit antibody (diluted 1/1000, A24541; Life Technologies, Carlsbad, CA, USA), chgA was detected with Alexa Fluor 568-conjugated Streptavidin (S11226; Invitrogen, Waltham, MA, USA). Sections were mounted using ProLong Gold Antifade Reagent (Invitrogen) containing DAPI to counterstain the nuclei. The density of CCK-producing cells that were stained green (endogenous GFP) and positive for chgA was measured by fluorescence microscopy (Axio Imager M2m; Zeiss, Iéna, Germany) in the crypts of three duodenal sections under a 40× objective. The data were expressed as the percentage of chgA-positive cells or CCK (GFP)-positive cells per crypt.

### 4.12. Sample Nomenclature

The nomenclature for identifying a sample is, stomach “sto”; brain stem “bs”; hippocampus “hip”; hypothalamus “hy”; weaning at Day-15 “w15”; weaning at Day-30 “w30”; oral bolus with miR-320-3p/DOSP “b320”; oral bolus with miR-375-3p/DOSP “b375”; and oral bolus of controls “btem”. For example, hy-w15-btem means that the rat sample is the sample obtained from the hypothalamus of a pup weaned at Day-15 having received a control bolus.

### 4.13. Statistical Analysis

The first litter (from one mother) with 12 rat pups was divided into three groups (*n* = 4 per group) and randomly assigned to one of the following treatments: miR-320-3p/DOSP, miR-375-3p/DOSP, and control (DMEM/PBS). The remaining eight litters were randomly assigned to control and treatment groups, with all rat pups of a litter receiving the same treatment. The samples were analyzed in parallel and blind design within months after the euthanizing of the last litters. The normality of distribution was tested by the Shapiro–Wilks test, and the delta-Cq data were improved by log transformation, justifying our use of ANOVA for the comparison between groups. Multiple Comparisons of Means (Tukey Contrasts) were also performed when needed between groups. The data on the expression of miR-320-3p, miR-375-3p, miR-16-5p, miR-132-3p, and miR-594 of four litters (miR-320-3p/DOSP and Early Weaning, DOSP vehicle and Early Weaning, miR-320-3p/DOSP and Regular Weaning, and DOSP vehicle and Regular Weaning) were analyzed according to the analysis of variance two-way with interaction. Data analysis was performed using R Commander and R suite or GraphPad software. Cytoscape was used to create networks of miRNA and mRNA significantly deregulated in our dataset.

## Figures and Tables

**Table 1:** List of significantly altered micro and messenger RNAs in the duodenum, brain stem, hippocampus, and hypothalamus of early weaned transgenic rat pups treated by a single oral supplement of miR-320-3p and sacrificed at Day-45. Note that the miR-375-5p level of w15-b320 was downregulated in comparison with w30-b320 (*p* = 0.02), and w30-btem (*p* = 0.04). No differences were found between genes assayed on the stomach. The orange or blue background underlines the up-or-down regulation of miRNA and mRNA, respectively.

Tissue	miRNA			mRNA						
	**miR-320-3p**	**miR-504**	**miR-16-5p**	**drd2**	**sert**	**5ht1b**	**cck**	**gfp**	**clock**	**per1**
Brain stem-Female	**Up**	**Up**	**Up**		**Down**		**Down**			
Hypothalamus-Female						**Up**			**Up**	**Up**
Hippocampus-Female		**Down**								
Hypothalamus-Male				**Up**			**Up**	**Up**	**Up**	
Hippocampus-Male		**Down**								
Duodenum	**Up**	**Not expressed**		**Not Done**	**Not Done**	**Not Done**	**Not Done**	**Not Done**	**Not Done**	**Not Done**

## Data Availability

Excel files with raw Cq data organized by tissues can be accessed at the UN-Cloud of Nantes University.

## Supplementary Materials

The following supporting information can be downloaded at: https://www.mdpi.com/article/10.3390/ijms24010191/s1.

## References

[1] Vaiserman A.M. (2015). Epigenetic programming by early-life stress: Evidence from human populations. Dev. Dyn..

[2] Allen L., Dwivedi Y. (2020). MicroRNA mediators of early life stress vulnerability to depression and suicidal behavior. Mol. Psychiatry.

[3] Tavares G.A., Torres A., De Souza J.A. (2020). Early Life Stress and the Onset of Obesity: Proof of MicroRNAs’ Involvement Through Modulation of Serotonin and Dopamine Systems’ Homeostasis. Front. Physiol..

[4] Huang W., Li M.D. (2009). Differential Allelic Expression of Dopamine D1 Receptor Gene (DRD1) Is Modulated by microRNA miR-504. Biol. Psychiatry.

[5] Shao Q.-Y., You F., Zhang Y.-H., Hu L.-L., Liu W.-J., Liu Y., Li J., Wang S.-D., Song M.-F. (2018). CSF miR-16 expression and its association with miR-16 and serotonin transporter in the raphe of a rat model of depression. J. Affect. Disord..

[6] Alvarez-Saavedra M., Antoun G., Yanagiya A., Oliva-Hernandez R., Cornejo-Palma D., Perez-Iratxeta C., Sonenberg N., Cheng H.-Y.M. (2011). miRNA-132 orchestrates chromatin remodeling and translational control of the circadian clock. Hum. Mol. Genet..

[7] Aten S., Hansen K.F., Price K.H., Wheaton K., Kalidindi A., Garcia A., Alzate-Correa D., Hoyt K.R., Obrietan K. (2018). miR-132 couples the circadian clock to daily rhythms of neuronal plasticity and cognition. Learn. Mem..

[8] Chen Q., Zhang F., Dong L., Wu H., Xu J., Li H., Wang J., Zhou Z., Liu C., Wang Y. (2021). SIDT1-dependent absorption in the stomach mediates host uptake of dietary and orally administered microRNAs. Cell Res..

[9] Kosaka N., Izumi H., Sekine K., Ochiya T. (2010). microRNA as a new immune-regulatory agent in breast milk. Silence.

[10] Lukasik A., Brzozowska I., Zielenkiewicz U., Zielenkiewicz P. (2017). Detection of Plant miRNAs Abundance in Human Breast Milk. Int. J. Mol. Sci..

[11] Ozkan H., Tuzun F., Taheri S., Korhan P., Akokay P., Yılmaz O., Duman N., Özer E., Tufan E., Kumral A. (2020). Epigenetic Programming Through Breast Milk and Its Impact on Milk-Siblings Mating. Front. Genet..

[12] Baier S.R., Nguyen C., Xie F., Wood J.R., Zempleni J. (2014). MicroRNAs Are Absorbed in Biologically Meaningful Amounts from Nutritionally Relevant Doses of Cow Milk and Affect Gene Expression in Peripheral Blood Mononuclear Cells, HEK-293 Kidney Cell Cultures, and Mouse Livers. J. Nutr..

[13] Wang L., Sadri M., Giraud D., Zempleni J. (2018). RNase H2-Dependent Polymerase Chain Reaction and Elimination of Confounders in Sample Collection, Storage, and Analysis Strengthen Evidence That microRNAs in Bovine Milk Are Bioavailable in Humans. J. Nutr..

[14] Chauhan N., Jaggi M., Chauhan S.C., Yallapu M.M. (2021). COVID-19: Fighting the invisible enemy with microRNAs. Expert Rev. Anti-Infect. Ther..

[15] del Pozo-Acebo L., Hazas M.L.D.L., Margollés A., Dávalos A., García-Ruiz A. (2021). Eating microRNAs: Pharmacological opportunities for cross-kingdom regulation and implications in host gene and gut microbiota modulation. Br. J. Pharmacol..

[16] Desvignes T., Batzel P., Berezikov E., Eilbeck K., Eppig J.T., McAndrews M.S., Singer A., Postlethwait J.H. (2015). miRNA Nomenclature: A View Incorporating Genetic Origins, Biosynthetic Pathways, and Sequence Variants. Trends Genet..

[17] Zhou Q., Li M., Wang X., Li Q., Wang T., Zhou X., Wang X., Gao X., Li X. (2012). Immune-related MicroRNAs are Abundant in Breast Milk Exosomes. Int. J. Biol. Sci..

[18] Beuzelin D., Kaeffer B. (2018). Exosomes and miRNA-Loaded Biomimetic Nanovehicles, a Focus on Their Potentials Preventing Type-2 Diabetes Linked to Metabolic Syndrome. Front. Immunol..

[19] Kim D.H., Sætrom P., Snøve O., Rossi J.J. (2008). MicroRNA-directed transcriptional gene silencing in mammalian cells. Proc. Natl. Acad. Sci. USA.

[20] Beuzelin D., Pitard B., Kaeffer B. (2019). Oral Delivery of miRNA With Lipidic Aminoglycoside Derivatives in the Breastfed Rat. Front. Physiol..

[21] Pierdomenico M., Cesi V., Cucchiara S., Vitali R., Prete E., Costanzo M., Aloi M., Oliva S., Stronati L. (2016). NOD2 Is Regulated By Mir-320 in Physiological Conditions but this Control Is Altered in Inflamed Tissues of Patients with Inflammatory Bowel Disease. Inflamm. Bowel Dis..

[22] He M., Wang J., Yin Z., Zhao Y., Hou H., Fan J., Li H., Wen Z., Tang J., Wang Y. (2019). MiR-320a induces diabetic nephropathy via inhibiting MafB. Aging.

[23] Li Y., Huang J., Hu C., Zhou J., Xu D., Hou Y., Wu C., Zhao J., Li M., Zeng X. (2021). MicroRNA-320a: An important regulator in the fibrotic process in interstitial lung disease of systemic sclerosis. Thromb. Haemost..

[24] Du H., Zhao Y., Yin Z., Wang D.W., Chen C. (2021). The role of miR-320 in glucose and lipid metabolism disorder-associated diseases. Int. J. Biol. Sci..

[25] Knudsen L.A., Petersen N., Schwartz T.W., Egerod K.L. (2015). The MicroRNA Repertoire in Enteroendocrine Cells: Identification of miR-375 as a Potential Regulator of the Enteroendocrine Lineage. Endocrinology.

[26] Van der Auwera S., Ameling S., Nauck M., Völzke H., Völker U., Grabe H.J. (2021). Association between different dimensions of childhood traumatization and plasma micro-RNA levels in a clinical psychiatric sample. J. Psychiatr. Res..

[27] Abdelmohsen K., Hutchison E.R., Lee E.K., Kuwano Y., Kim M.M., Masuda K., Srikantan S., Subaran S.S., Marasa B.S., Mattson M.P. (2010). miR-375 Inhibits Differentiation of Neurites by Lowering HuD Levels. Mol. Cell. Biol..

[28] Alles J., Fehlmann T., Fischer U., Backes C., Galata V., Minet M., Hart M., Abu-Halima M., Grässer F.A., Lenhof H.-P. (2019). An estimate of the total number of true human miRNAs. Nucleic Acids Res..

[29] Gapp K., Jawaid A., Sarkies P., Bohacek J., Pelczar P., Prados J., Farinelli L., Miska E., Mansuy I.M. (2014). Implication of sperm RNAs in transgenerational inheritance of the effects of early trauma in mice. Nat. Neurosci..

[30] Lotan A., Lifschytz T., Wolf G., Keller S., Ben-Ari H., Tatarsky P., Pillar N., Oved K., Sharabany J., Merzel T.K. (2018). Differential effects of chronic stress in young-adult and old female mice: Cognitive-behavioral manifestations and neurobiological correlates. Mol. Psychiatry.

[31] Denk J., Boelmans K., Siegismund C.S., Lassner D., Arlt S., Jahn H. (2015). MicroRNA profiling of CSF reveals potential biomarkers to detect Alzheimer`s disease. PLoS ONE.

[32] Desigaux L., Sainlos M., Lambert O., Chevre R., Letrou-Bonneval E., Vigneron J.-P., Lehn P., Lehn J.-M., Pitard B. (2007). Self-assembled lamellar complexes of siRNA with lipidic aminoglycoside derivatives promote efficient siRNA delivery and interference. Proc. Natl. Acad. Sci. USA.

[33] Mével M., Haudebourg T., Colombani T., Peuziat P., Dallet L., Chatin B., Lambert O., Berchel M., Montier T., Jaffrès P.-A. (2016). Important role of phosphoramido linkage in imidazole-based dioleyl helper lipids for liposome stability and primary cell transfection. J. Gene Med..

[34] Habrant D., Peuziat P., Colombani T., Dallet L., Gehin J., Goudeau E., Evrard B., Lambert O., Haudebourg T., Pitard B. (2016). Design of Ionizable Lipids To Overcome the Limiting Step of Endosomal Escape: Application in the Intracellular Delivery of mRNA, DNA, and siRNA. J. Med. Chem..

[35] Colombani T., Peuziat P., Dallet L., Haudebourg T., Mével M., Berchel M., Lambert O., Habrant D., Pitard B. (2017). Self-assembling complexes between binary mixtures of lipids with different linkers and nucleic acids promote universal mRNA, DNA and siRNA delivery. J. Control. Release.

[36] Le Gall T., Berchel M., Davies L., Mottais A., Ghanem R., Fautrel A., Gill D., Hyde S., Lehn P., Lehn J.-M. (2021). Aerosol-Mediated Non-Viral Lung Gene Therapy: The Potential of Aminoglycoside-Based Cationic Liposomes. Pharmaceutics.

[37] Olejniczak M., Galka P., Krzyzosiak W.J. (2010). Sequence-non-specific effects of RNA interference triggers and microRNA regulators. Nucleic Acids Res..

[38] Sticht C., De La Torre C., Parveen A., Gretz N. (2018). miRWalk: An online resource for prediction of microRNA binding sites. PLoS ONE.

[39] Shalev-Benami M., Zhang Y., Rozenberg H., Nobe Y., Taoka M., Matzov D., Zimmerman E., Bashan A., Isobe T., Jaffe C.L. (2017). Atomic resolution snapshot of Leishmania ribosome inhibition by the aminoglycoside paromomycin. Nat. Commun..

[40] Garcia-Martin R., Wang G., Brandão B.B., Zanotto T.M., Shah S., Patel S.K., Schilling B., Kahn C.R. (2022). MicroRNA sequence codes for small extracellular vesicle release and cellular retention. Nature.

[41] Oluwaseun Adetunji C., Samuel Michael O., Rathee S., Singh K.R.B., Olufemi Ajayi O., Bunmi Adetunji J., Ojha A., Singh J., Singh R.P. (2022). Potentialities of nanomaterials for the management and treatment of metabolic syndrome: A new insight. Mater. Today Adv..

[42] Amiri A., Bagherifar R., Dezfouli E.A., Kiaie S.H., Jafari R., Ramezani R. (2022). Exosomes as bio-inspired nanocarriers for RNA delivery: Preparation and applications. J. Transl. Med..

[43] Groot M., Lee H. (2020). Sorting Mechanisms for MicroRNAs into Extracellular Vesicles and Their Associated Diseases. Cells.

[44] McKibben L.A., Dwivedi Y. (2021). Early life and adult stress promote sex dependent changes in hypothalamic miRNAs and environmental enrichment prevents stress-induced miRNA and gene expression changes in rats. BMC Genom..

[45] Zhang Y., Wang Y., Wang L., Bai M., Zhang X., Zhu X. (2015). Dopamine Receptor D2 and Associated microRNAs Are Involved in Stress Susceptibility and Resistance to Escitalopram Treatment. Int. J. Neuropsychopharmacol..

[46] Wu X., Xu F.-L., Xia X., Wang B.-J., Yao J. (2020). MicroRNA-15a, microRNA-15b and microRNA-16 inhibit the human dopamine D1 receptor expression in four cell lines by targeting 3′UTR –12 bp to + 154 bp. Artif. Cells Nanomed. Biotechnol..

[47] Rincel M., Olier M., Minni A., de Oliveira C.M., Matime Y., Gaultier E., Grit I., Helbling J.-C., Costa A.M., Lépinay A. (2019). Pharmacological restoration of gut barrier function in stressed neonates partially reverses long-term alterations associated with maternal separation. Psychopharmacology.

[48] Coupé B., Amarger V., Grit I., Benani A., Parnet P. (2010). Nutritional Programming Affects Hypothalamic Organization and Early Response to Leptin. Endocrinology.

[49] Linsen S.E., de Wit E., de Bruijn E., Cuppen E. (2010). Small RNA expression and strain specificity in the rat. BMC Genom..

[50] Young C., Caffrey M., Janton C., Kobayashi T. (2022). Reversing the miRNA -5p/-3p stoichiometry reveals physiological roles and targets of miR-140 miRNAs. RNA.

[51] Pomar C.A., Serra F., Palou A., Sánchez J. (2021). Lower miR-26a levels in breastmilk affect gene expression in adipose tissue of offspring. FASEB J..

[52] Yarani R., Shojaeian A., Palasca O., Doncheva N.T., Jensen L.J., Gorodkin J., Pociot F. (2022). Differentially Expressed miRNAs in Ulcerative Colitis and Crohn’s Disease. Front. Immunol..

[53] Yeganeh M., Hernandez N. (2020). RNA polymerase III transcription as a disease factor. Genes Dev..

[54] Kulaberoglu Y., Malik Y., Borland G., Selman C., Alic N., Tullet J.M.A. (2021). RNA Polymerase III, Ageing and Longevity. Front. Genet..

[55] Montecalvo A., Larregina A.T., Shufesky W.J., Beer Stolz D., Sullivan M.L.G., Karlsson J.M., Baty C.J., Gibson G.A., Erdos G., Wang Z. (2012). Mechanism of transfer of functional microRNAs between mouse dendritic cells via exosomes. Blood.

[56] Liu Y., Lin J., Tu H., Chang C. (2015). Protecting against ischemic stroke in rats by heat shock protein-20-mediated exercise preconditioning. FASEB J..

[57] Salmena L., Poliseno L., Tay Y., Kats L., Pandolfi P.P. (2011). A ceRNA Hypothesis: The Rosetta Stone of a Hidden RNA Language?. Cell.

[58] Ip S., Chung M., Raman G., Chew P., Magula N., Devine D., Trikalinos T., Lau J. (2007). Breastfeeding and maternal and infant health outcomes in developed countries. Evid. Rep. Technol. Assess..

[59] Ferrero G., Carpi S., Polini B., Pardini B., Nieri P., Impeduglia A., Grioni S., Tarallo S., Naccarati A. (2021). Intake of Natural Compounds and Circulating microRNA Expression Levels: Their Relationship Investigated in Healthy Subjects With Different Dietary Habits. Front. Pharmacol..

[60] Orozco-Solís R., Matos R.J.B., De Souza S.L., Grit I., Kaeffer B., De Castro R.M., Bolaños-Jiménez F. (2011). Perinatal nutrient restriction induces long-lasting alterations in the circadian expression pattern of genes regulating food intake and energy metabolism. Int. J. Obes..

[61] Tavares G.A., Almeida L.C.D.A., de Souza J.A., de Farias V.V., de Souza F.L., Silva S.C.D.A., Lagranha C.J., Kaeffer B., de Souza S.L. (2020). Early weaning leads to disruption of homeostatic and hedonic eating behaviors and modulates serotonin (5HT) and dopamine (DA) systems in male adult rats. Behav. Brain Res..

[62] Ménoret S., De Cian A., Tesson L., Remy S., Usal C., Boulé J.-B., Boix C., Fontanière S., Crénéguy A., Nguyen T.H. (2015). Homology-directed repair in rodent zygotes using Cas9 and TALEN engineered proteins. Sci. Rep..

[63] Ndjim M., Meriaux R., Falcon L., Segain J.-P., Remy S., Tesson L., Le Drean G. Maternal protein restriction increases duodenal entroendocrine cells in young rats. Proceedings of the 9. World Congress Developmental Origins of Health and Disease (DOHAD).

[64] Shimoyama M., Hayman G.T., Laulederkind S.J.F., Nigam R., Lowry T.F., Petri V., Smith J.R., Wnge S.-J., Munzenmaier D.H., Dwinell M.R. (2009). The Rat Genome Database Curators: Who, What, Where, Why. PLoS Comput. Biol..

[65] Stevanato L., Thanabalasundaram L., Vysokov N., Sinden J.D. (2016). Investigation of Content, Stoichiometry and Transfer of miRNA from Human Neural Stem Cell Line Derived Exosomes. PLoS ONE.

[66] Roberts T.C. (2014). The MicroRNA Biology of the Mammalian Nucleus. Mol. Ther.-Nucleic Acids.

[67] Kalantari R., Chiang C.-M., Corey D.R. (2016). Regulation of mammalian transcription and splicing by Nuclear RNAi. Nucleic Acids Res..

