# Supplementary material for: Oral Delivery of miR-320-3p with Lipidic Aminoglycoside Derivatives at Mid-Lactation Alters miR-320-3p Endogenous Levels in the Gut and Brain of Adult Rats According to Early or Regular Weaning
Source: Int J Mol Sci. 2022 Dec 22;24(1):191. doi: 10.3390/ijms24010191 (PMC9820440; doi:10.3390/ijms24010191)
Supplement: Supplementary file 1 [file ijms-24-00191-s001.zip › ijms-2089160-supplementary.pdf]

Supplementary Materials:

Article

Oral Delivery of miR-320-3p with Lipidic Aminoglycoside Derivatives at Mid-Lactation Alters miR-320-3p Endogenous Levels in the Gut and Brain of Adult Rats According to Early or Regular Weaning

Gabriel Araujo Tavares [1,2,†,‡], Amada Torres [1,†], Gwenola Le Drean [1], Maïwenn Queignec [1], Blandine Castellano [1], Laurent Tesson [3], Séverine Remy [3], Ignacio Anegon [3], Bruno Pitard [4] and Bertrand Kaeffer [1,*]

Supplementary Materials: Excel files of raw Cq data organized by tissues can be made accessible at the UN-Cloud of the University of Nantes.

Table-S1: miR Pathway enrichment analysis for rno-miR-320-3p and rno-miR-375-3p. To identify pathways from the list of the potential regulated mRNA we used Panther v 16.0 (released 2020-12-01, http://www.pantherdb.org/). To ensure the validity of our findings, we only considered the three pathways more relevant on both miRNAs.

The potential 111 co-regulated transcript by miR-320-3p are distribute between 69 pathways, while the 24 candidates genes associated to miR-375-3p were found in 12 pathways. Interesting, both miRs present the pathways of: a) Inflammation mediated by chemokines and cytokines signaling pathway, b) Interferon-gamma signaling pathway and c) Wnt signaling pathway, with different interacting genes.

A. Result of PANTHER Full list of pathway related with miR-320-3p

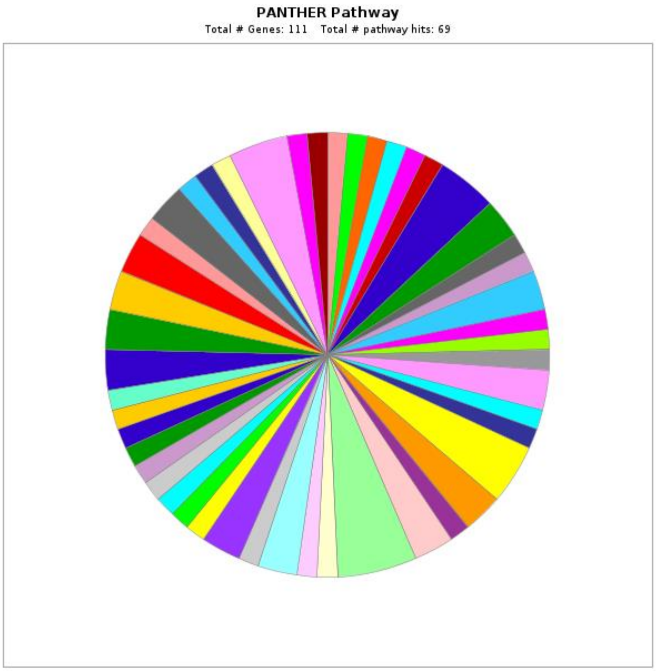

Click to get gene list for a category:

5HT1 type receptor mediated signaling pathway (P04373)
Alzheimer disease-amyloid secretase pathway (P00003)
Angiogenesis (P00005)
Angiotensin II-stimulated signaling through G proteins and beta-arrestin (P05911)
Apoptosis signaling pathway (P00006)
B cell activation (P00010)
CCKR signaling map (P06959)
Cadherin signaling pathway (P00012)
Cytoskeletal regulation by Rho GTPase (P00016)
Dopamine receptor mediated signaling pathway (P05912)
EGF receptor signaling pathway (P00018)
Endogenous cannabinoid signaling (P05730)
Endothelin signaling pathway (P00019)
Enkephalin release (P05913)

**
Chart tooltips are read as: Category name (Accession): # genes; Percent of gene hit against total # genes; Percent of gene hit against total # Pathway hits

FGF signaling pathway (P00021)

GABA-B receptor II signaling (P05731)

Glycolysis (P00024)

Gonadotropin-releasing hormone receptor pathway (P06664)

Heterotrimeric G-protein signaling pathway-Gi alpha and Gs alpha mediated pathway (P00026)

Heterotrimeric G-protein signaling pathway-Gq alpha and Go alpha mediated pathway (P00027)

Huntington disease (P00029)

Inflammation mediated by chemokine and cytokine signaling pathway (P00031)

Insulin/IGF pathway-mitogen activated protein kinase kinase/MAP kinase cascade (P00032)

Insulin/IGF pathway-protein kinase B signaling cascade (P00033)

Integrin signalling pathway (P00034)

Interferon-gamma signaling pathway (P00035)

Interleukin signaling pathway (P00036)

Metabotropic glutamate receptor group II pathway (P00040)

Metabotropic glutamate receptor group III pathway (P00039)

Muscarinic acetylcholine receptor 2 and 4 signaling pathway

(P00043) Nicotine pharmacodynamics pathway (P06587)

Nicotinic acetylcholine receptor signaling pathway (P00044)

Opioid prodynorphin pathway (P05916)

Opioid proenkephalin pathway (P05915)

Opioid proopiomelanocortin pathway (P05917)

Ornithine degradation (P02758)

PDGF signaling pathway (P00047)

PI3 kinase pathway (P00048)

Parkinson disease (P00049)

Ras Pathway (P04393)

T cell activation (P00053)

TGF-beta signaling pathway (P00052)

Toll receptor signaling pathway (P00054)

Ubiquitin proteasome pathway (P00060)

VEGF signaling pathway (P00056)

Wnt signaling pathway (P00057)

p53 pathway feedback loops 2 (P04398)

p53 pathway (P00059)

B. Result of PANTHER Full list of pathway related with miR-375-3p

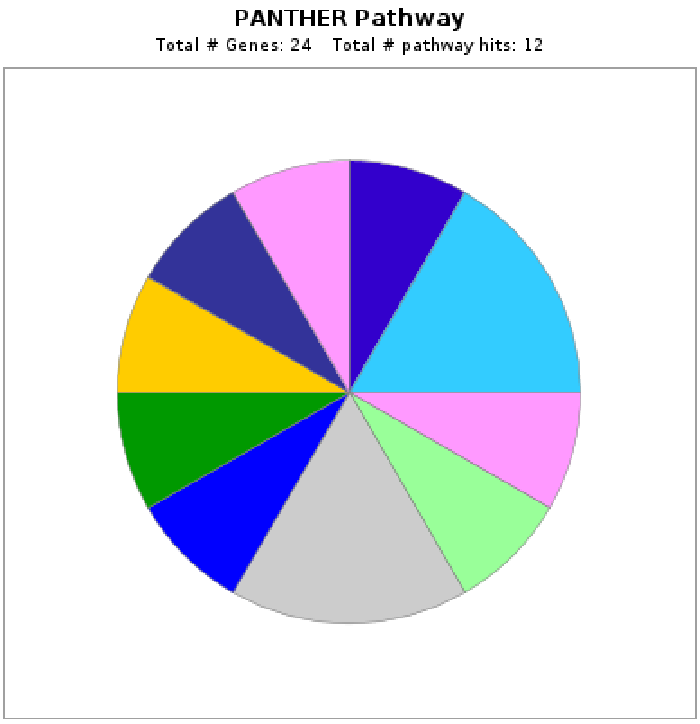

Click to get gene list for a category:

- CCKR signaling map (P06959)
- EGF receptor signaling pathway (P00018)
- FGF signaling pathway (P00021)
- Inflammation mediated by chemokine and cytokine signaling pathway (P00031)
- Interferon-gamma signaling pathway (P00035)
- JAK/STAT signaling pathway (P00038)
- PI3 kinase pathway (P00048)
- Parkinson disease (P00049)
- Ubiquitin proteasome pathway (P00060)
- Wnt signaling pathway (P00057)

Table S2. Details of the primers used to construct the transgenic rats, and quantified 9 miRNA and 38 mRNA.

**Gene Name**	**Primer Sequence**	**Reference**
rROSA-fw1	TGAACTGTGAATAGGCCCAAGTG	[62]
rROSA-rev1	GCATTTTAAAAGAGCCCAGTACTTCA	[62]
GFP Up	CCTCGTGACCACCCTGACCT	[62]
GFP Lo3	TCCATGCCGAGAGTGATCCC	[62]
rROSA26-5outFor	TCCCACCCTCCCCTTCCTCT	[62]
5rCCKpRev	TGTGACCCCGTTGCCCTGGAT	[62]
3BGHpA-Up2	CCAGATTTTTCCTCCTCTCCTG	[62]
rROSA26-3outRev	TGGGTATCACTGGCTGTCCTAGATA	[62]
microRNAs	TaqMan System	
Let-7d-5p	Advanced-rno478439_mir	
Let-7g-5p	Advanced-rno478580_mir	
rno-miR-132-3p	Advanced-rno480919_mir	
rno-miR-146b	Advanced-rno480941_mir	
rno-miR-16-5p	Advanced rno481312_mir	
rno-miR-320-3p	Advanced-rno481048_mir	
rno-miR-375-3p	Advanced-rno481142_mir	
rno-miR-375-5p	Advanced-rno481142_mir	
rno-miR-504	Advanced-rno481198_mir	
microRNAs	SYBR-Green System	
miR-375-5p-fw	GCGACGAGCCCCUCGCACAAACC	
Universal miRNA reverse	GCAGGGTCCGAGGTATTC	[68]
β2-microglobulin	rno0560865_m1	
β-actin	rno0667869_m1	
usb1	rn01536722_m1	
Polr3d	rno1468090_g1	
Hspb6	rno0577590_ml	
SERT	rno00564737_mir	
	MESSENGER for SYBR-Green System	
GAPDH2-fw	CGG CAA GTT CAA CGG CAC AG	
GAPDH2-rv	TCC ACG ACA TAC TCA TCA GCA CCA	

β-actin-fw	CTA TCG GCA ATG AGC GGT TCC
β-actin-rv	GCA CTG TGT TGG CAT AGA GGT C
β -2M-fw	TGA CCG TGA TCT TTC TGG TG
β -2M-rv	ACT TGA ATT TGG GGA GTT TTC TG
	REPORTER GENES
GFP-fw	AAG CTG ACC CTG AAG TTC ATC TGC
GFP-rv	CTT GTA GTT GCC GTC GTC CTT GAA
CCK-fw	TGC TTG GAG GAG GCG GAA TG
CCK-rv	GCT GGG CTG AGG TGT GTG G
	PROMOTERS
POLR3D-fw	CAGACCAGTCACCTCATCCTTT
POLR3D-rv	AGTATTTATCAGACGGTGCCTC
MARF1-fw	GATAACCCCCTATTTTGAGGTT
MARF1-rv	GCGTCTTCTCCGCGCAGGGCAT
	FUNCTIONAL GENES
Rghrl -fw	AGAGGCGCCAGCTAACAAGTAA
Rghrl - rev	GCAGGAGAGTGCTGGGAGTT
rGip-fw	CTCCTGTTCCTGGCTGTC
rGip-rev	GGCGATGCTGTAATCACTG
rPYY-fw	AGCGGTATGGGAAAAGAGAAGTC
rPYY-rev	ACCACTGGTCCACACCTTCTG
rCCK-fw	GCCGCCTGCCCTCAAC
rCCK-rev	ACACACGCCGCACTTCATATC
rPax6-fw	ATACCTACACCCCTCCGCAC
rPax6-rev	TGAGTCCTGTTGAAGTGGTTCC
rPAX4-fw	GGATACACTGGGAGCCTTGTC
rPAX4-rev	GGATACACTGGGAGCCTTGTC
rFoxa1-fw	GTTCCGCACAGGGTTGGATA
rFoxa1-rev	CTG ACC GGG ACA GAG GAG TA
r Chga-fw	TACCCAATCACCAACCAGCC
r Chga 1 rev	TGAGACTCCGACTGACCATC

5HT1B-fw	AGA AGA AAC TCA TGG CCG CT
5HT1B-rv	GGG GAG CCA GCA GAG AAT AA
5HT2C-fw	ATT TGT GCC CCG TCT TGG ATT
5HT2C-rv	CGC GAA TTG AAC CGG CTA TG
BMAL1-fw	GAC TTC GCC TCC ACC TGT TC
BMAL1-rv	CAT TGT CTG GTT CAC TGT CTT CG
CLOCK-fw	GAA CTT GGC GTT GAG GAG TCT
CLOCK-rv	GTG ATC GAA CCT TTC CCA GTG C
DRD1-fw	GTT TGT GTG GT TGG GTG GG
DRD1-rv	GCT CAT GGT GGC TGG AAA AC
DRD2-fw	GAG CCA ACC TGA AGA GAC CA
DRD2-rv	GCA TCC ATT CTC CGC CTG TT
IFNg-fw	GAT CCA GCA CAA AGC TGT CA
IFNg-rv	GAC TCC TTT TCC GCT TCC TT
IL10-fw	GCA GTA GAG CAG GTG AAG AAT G
IL10-rv	CAG TAG ACG CCG GGT GGT TC
IL1α -fw	AAGACAAGCCTGTGTTGCTGAAGG
IL1α-rv	TCCCAGAAGAAAATGAGGTCGGTC
IL6-fw	TCC TAC CCC AAC TTC CAA TGC TC
IL6-rv	TTG GAT GGT CTT GGT CCT TAG GG
iNOS-fw	GAT TTT TCA CGA CAC CCT
iNOS-rv	GGT CCT CTG GTC AAC CTC
PERIOD1-fw	GCC CTG CTG CCT GCT CAT TG
PERIOD1-rv	AAC TTG GTG TGT GCC GTG GG
PERIOD2-fw	GCA CGC TGG CAA CCT TGA AG
PERIOD2-rv	GGC TGG CTC TCA CTG GAC ATT AG
STAT1-fw	AGG TCC GTC AGC AGC TTA AA
STAT1-rv	CGA TCG GAT AAC AAC TGC TT
TGFβ-fw	AGT GGC TGT TGC GGA GAG
TGFβ-rv	GCT GAA AGG TAT GAC ATG GAC A
TNFα-fw	AAATGGGCTCCCTCTCATCAGTTC
TNFα-rv	TCTGCTTGGTGGTTTGCTACC
SIDT-1-fw	CGTCATCCGGACCAAGATGT
SIDT-1-rev	AGATGTCCTGGTTGCCAGTG

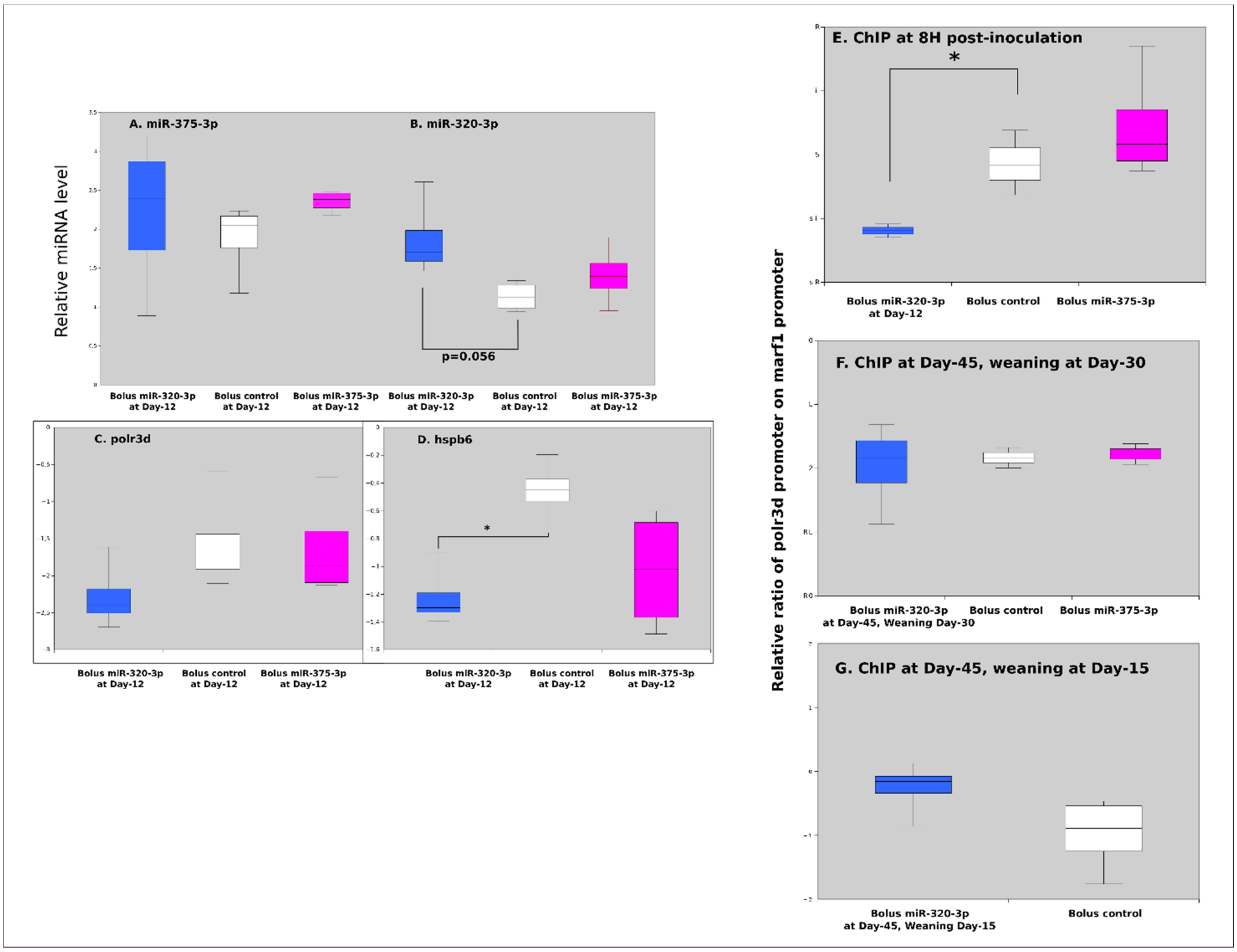

Figure S1. Immediate effect of miRNA supplementation. Evolution of miR-375-3p (A), 320-3p (B), polr3d (C) and hspb6 (D) mRNA, 8 hours after bolus for rat pups at Day-12 in stomach wall. Chromatin-immunoprecipitation assay against H3K4me3. Note the significant alteration at 8H after a bolus with miR-320-3p in gastric cells (E) and the absence of memory effect after regular (F) or early (G) weaning. The light gray background reminds that rats were sacrificed in the dark phase. Note: * $p<0.05$; ** $p<0.01$; *** $p<0.001$.

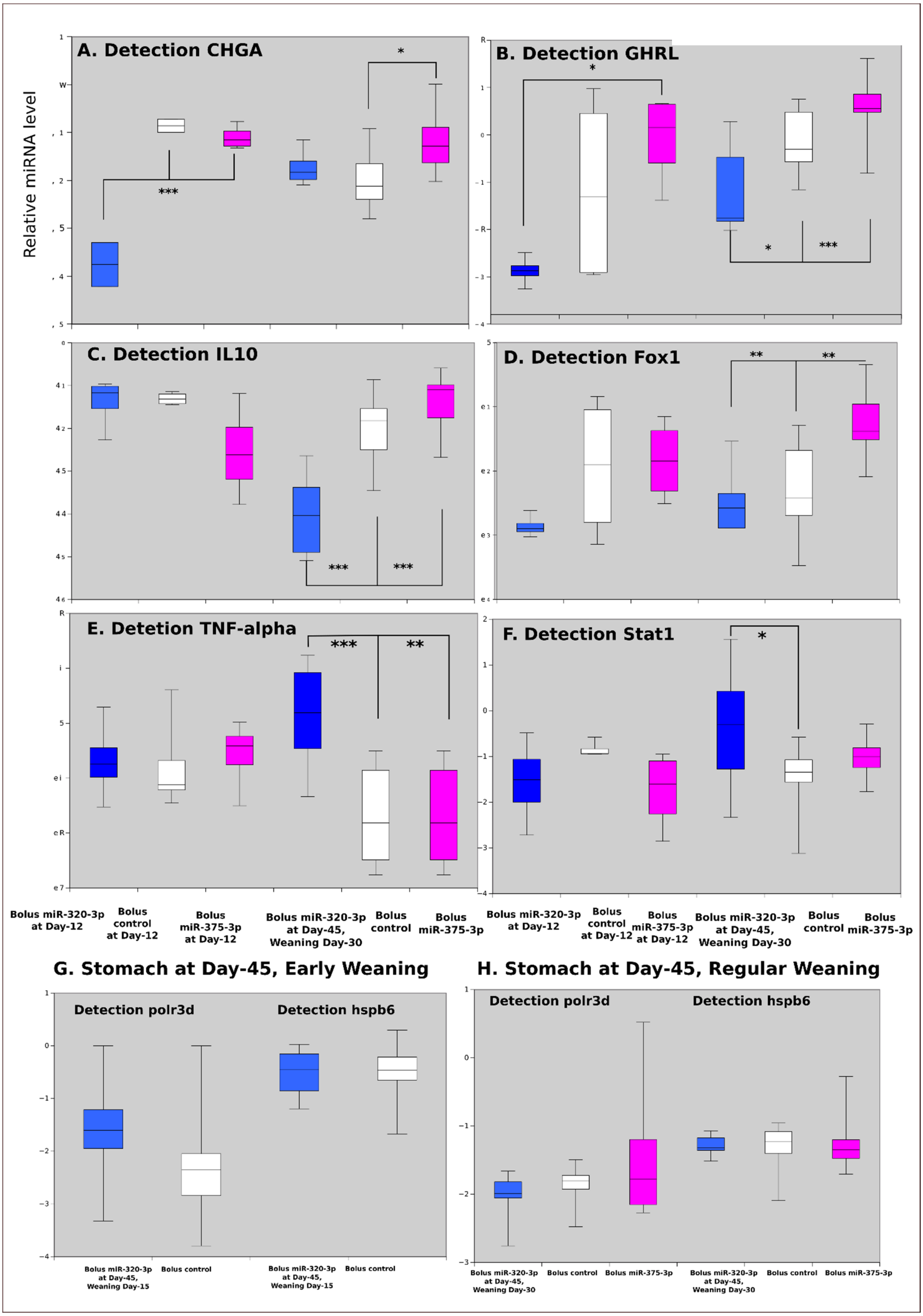

Relative miRNA level
A. Detection CHGA
B. Detection GHRL
C. Detection IL10
D. Detection Fox1
E. Detetion TNF-alpha
F. Detection Stat1
Bolus miR-320-3p at Day-12
Bolus control at Day-12
Bolus miR-375-3p at Day-12
Bolus miR-320-3p at Day-45, Weaning Day-30
Bolus control
Bolus miR-375-3p
G. Stomach at Day-45, Early Weaning
H. Stomach at Day-45, Regular Weaning
Detection polr3d
Detection hspb6
Bolus miR-320-3p at Day-45, Weaning Day-15
Bolus control
Bolus miR-320-3p at Day-45, Weaning Day-30
Bolus miR-375-3p

Figure S2. Evolution of polr3d and hspb6 mRNAs in stomach wall,at Day-45 after early (A) or regular (B) weaning. Concerning the inflammation status, the IL-10 [C], Fox1 (D), ChGRA (E), and GHRL (F) transcripts were all down-regulated at Day-45 for rat treated with miR-320-3p/DOSP. The levels of polr3d and hspb6 were not different after early (G) or regular (H) weaning. The light gray background reminds that rats were sacrificed in the dark phase. Note: * $p<0.05$; ** $p<0.01$; *** $p<0.001$.

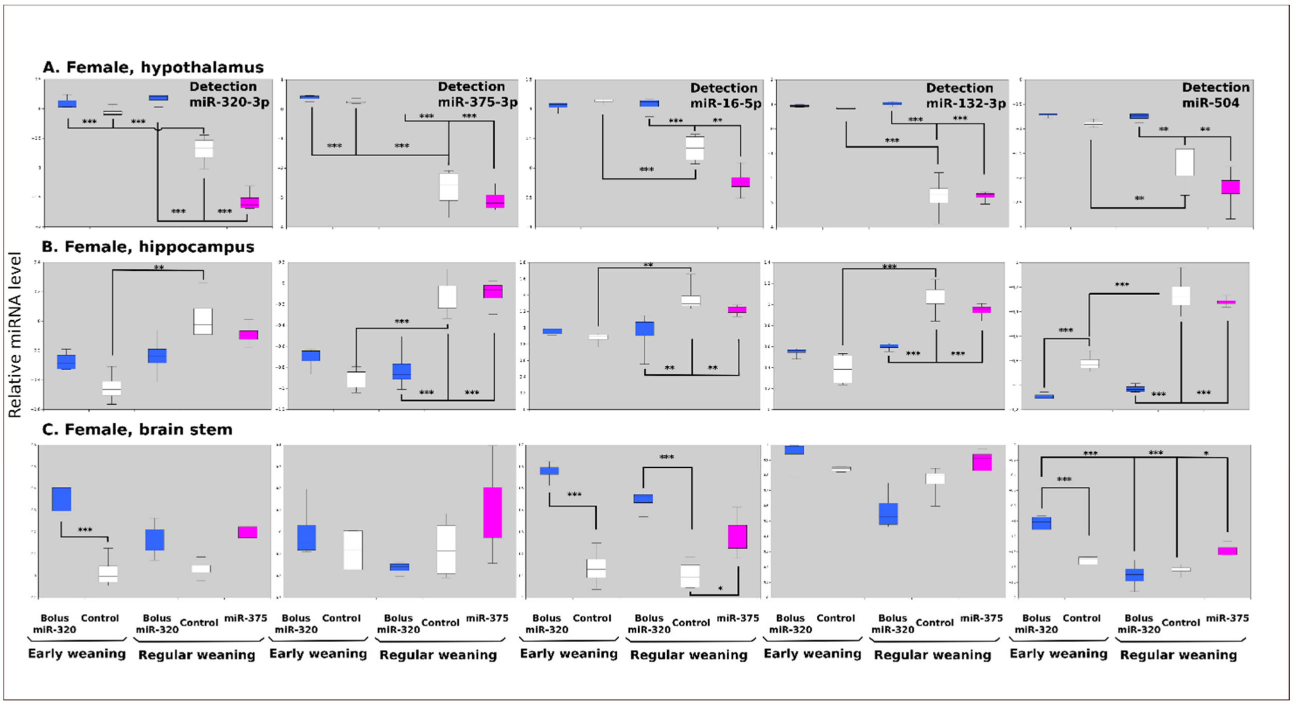

Figure S3. Evolution of miRNAs in hypothalamus (A), in hippocampus (B), and in brain stem (C) of females treated with miR-320-3p or 375-3p/DOSP according to early or regular weaning. The light gray background reminds that rats were sacrificed in the dark phase. Note: * p<0.05; ** p<0.01; *** p<0.001.

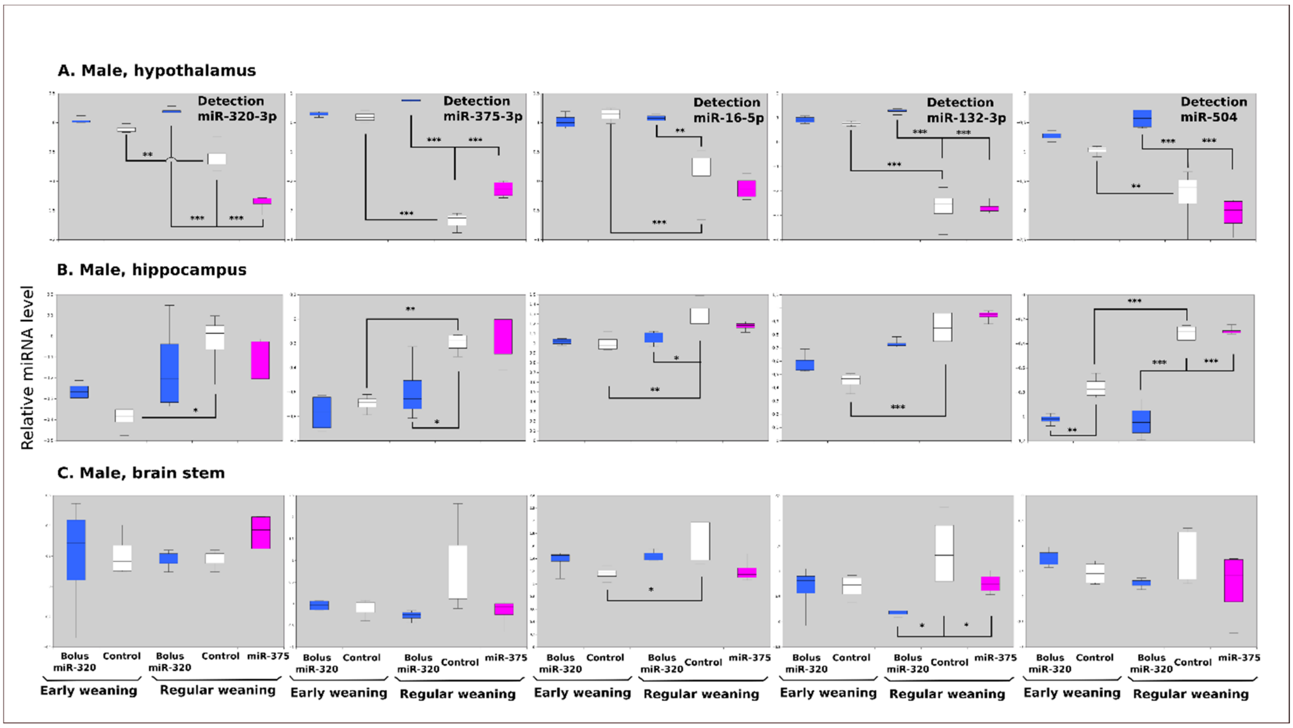

Figure S4. Evolution of miRNAs in hypothalamus (A), in hippocampus (B), and in brain stem (C) of males treated with miR-320-3p or 375-3p/DOSP according to early or regular weaning. The light gray background reminds that rats were sacrificed at ZT-20H in the dark phase. Note: * p<0.05; ** p<0.01; *** p<0.001.

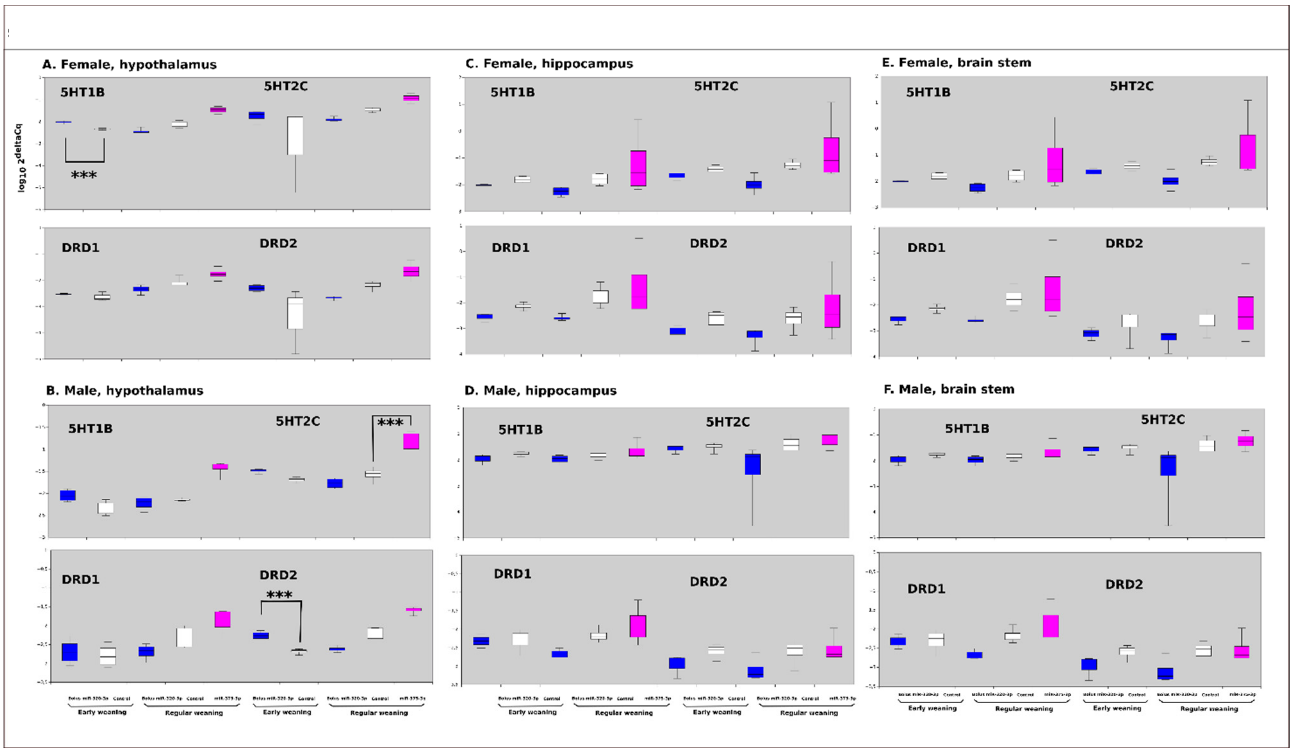

Figure S5. Evolution of transcripts related to serotonin and dopaminergic balance (5HT1B, 5HT2C, DRD1, DRD2) in hypothalamus (A, female ; B, male), in hippocampus (C, female ; D, male), and in brain stem (E, female, F, male) of rat treated with miR-320-3p or 375-3p/DOSP according to early or regular weaning. The light gray background reminds that rats were sacrificed in the dark phase. Note: * $p<0.05$; ** $p<0.01$; *** $p<0.001$.

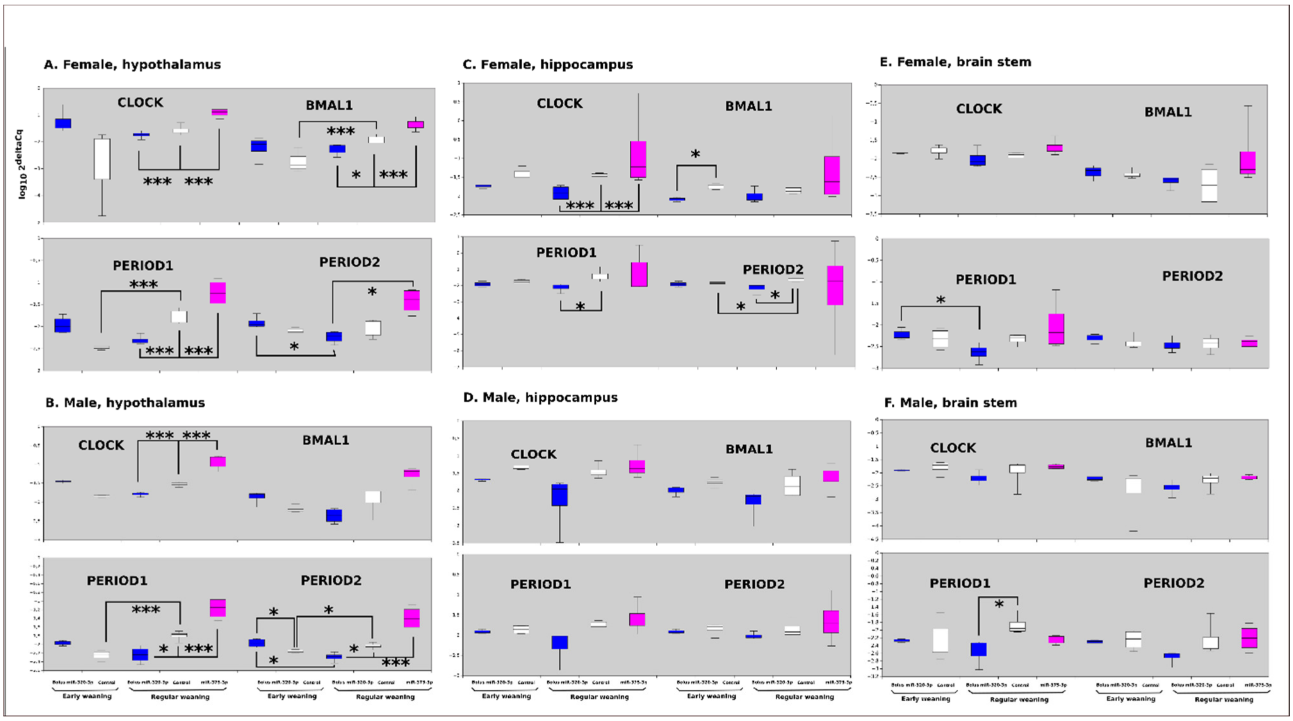

Figure S6. Evolution of transcripts related to the circadian clock (clock, bmal1, period1, period2) in hypothalamus (A, female ; B, male), in hippocampus (C, female ; D, male), and in brain stem (E, female, F, male) of rat treated with miR-320-3p or 375-3p/DOSP according to early or regular weaning. The light gray background reminds that rats were sacrificed in the dark phase. Note: * p<0.05; ** p<0.01; *** p<0.001.

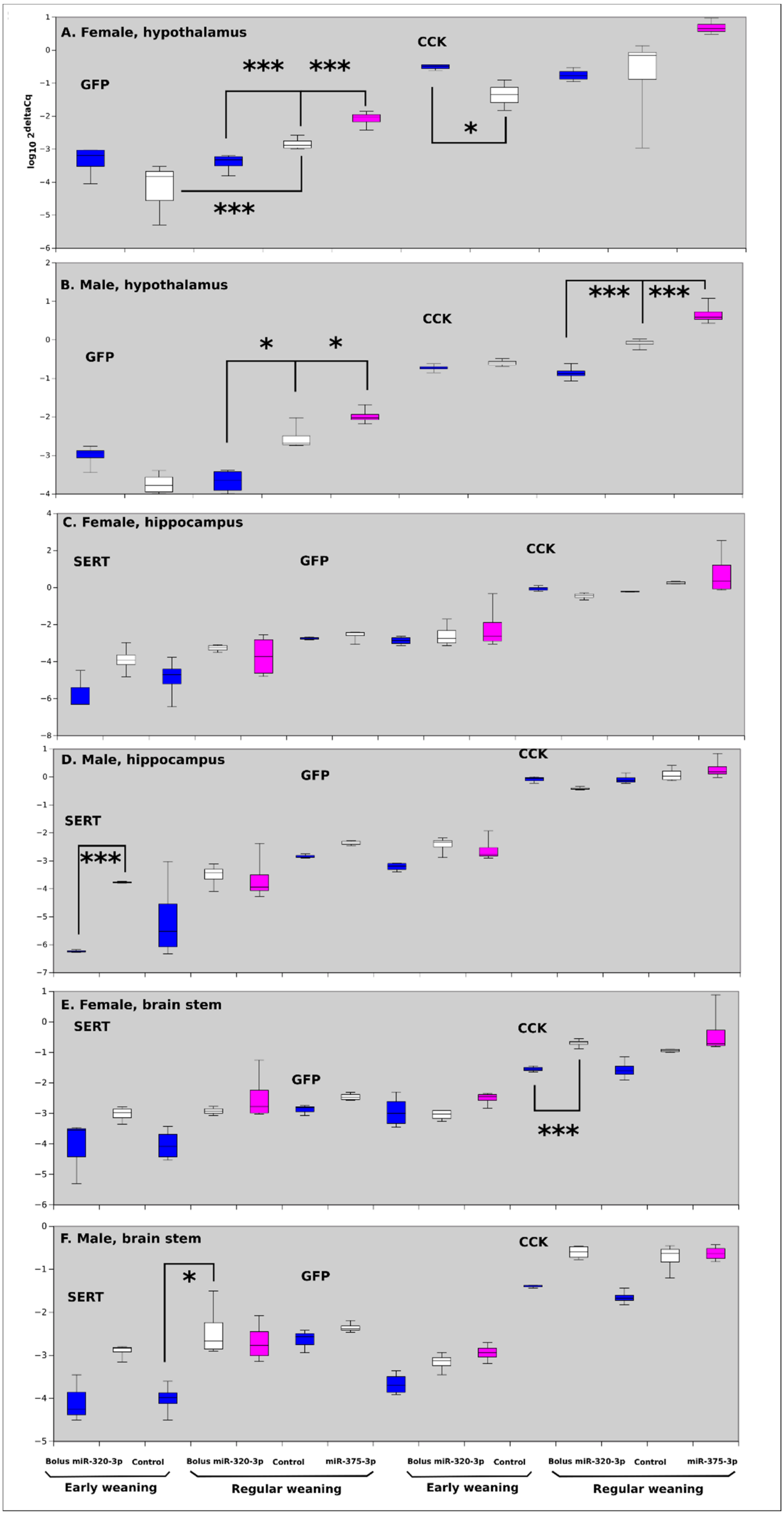
A. Female, hypothalamus
B. Male, hypothalamus
C. Female, hippocampus
D. Male, hippocampus
E. Female, brain stem
F. Male, brain stem
GFP
CCK
SERT
log10 2deltaCq
Bolus miR-320-3p
Control
miR-375-3p
Early weaning
Regular weaning

Figure S7. Evolution of SERT, GFP, CCK transcripts in hypothalamus (A, female ; B, male), in hippocampus (C, female ; D, male), and in brain stem (E, female, F, male) of rat treated with miR-320-3p or 375-3p/DOSP according to early or regular weaning. The light gray background reminds that rats were sacrificed in the dark phase. Note: * $p<0.05$; ** $p<0.01$; *** $p<0.001$.
